# Prior Expectations Bias Confidence Judgments Through Parietal Alpha‐Band Modulation

**DOI:** 10.1002/advs.202519417

**Published:** 2026-05-23

**Authors:** Luca Tarasi, Anna Pasini, Domenico Romanazzi, Margherita Covelli, Vincenzo Romei

**Affiliations:** ^1^ Centro Studi e Ricerche in Neuroscienze Cognitive Dipartimento di Psicologia “Renzo Canestrari”, Campus di Cesena, Alma Mater Studiorum – Università di Bologna Cesena Italy; ^2^ Universidad Antonio de Nebrija Madrid Spain

**Keywords:** alpha oscillations, confidence, metacognitive bias, prior expectations, TMS

## Abstract

Humans possess the metacognitive ability to estimate the likely accuracy of their own decisions through confidence judgments. Yet, whether prior information shapes confidence and the neural mechanisms mediating such influence, remain to be determined. Here, two complementary studies identify parietal alpha modulation as an oscillatory mechanism linking prior expectations to selective shifts in metacognitive bias. In Study 1, 75 participants performed a visual detection task with symbolic cues signaling target probability. Within the Type II Signal Detection Theory framework, probabilistic cues induce a congruence‐dependent metacognitive bias: confidence increases when perceptual responses align with expectations and decreases when they diverge. Importantly, metacognitive sensitivity is unaffected. Critically, electroencephalography shows that cue‐induced right‐parietal alpha modulation predicts interindividual differences in the magnitude of this bias. In Study 2 (N = 88), right‐parietal continuous Theta‐Burst Stimulation (cTBS) disrupts cue‐induced alpha modulation, relative to sham stimulation. This oscillatory change, in turn, drives the selective reduction in metacognitive bias. Together, these findings demonstrate that parietal alpha modulation causally and selectively shapes metacognitive bias, revealing an oscillatory mechanism for predictive metacognition.

## Abstract 2

When people expect something, they become more confident about having seen it, without any change in their ability to distinguish correct from incorrect decisions. Combining brain recordings and brain stimulation across two studies, the authors show that parietal alpha rhythms drive this effect, and that disrupting them eliminates the expectation‐driven confidence shift, linking brain oscillations to confidence bias.

## 1 Introduction

Perception is widely described as an integrative mechanism, where expectations merge with sensory evidence to build a coherent experience of reality [1]. Within this framework, perceptual decisions emerge from this interaction as the brain's optimal inference based on the environment's available information. Yet the perceptual process does not end with this initial decision. Observers can also estimate the likely accuracy of their decisions, reflecting a broader metacognitive capacity to evaluate their own cognitive operations across domains [2, 3, 4]. In the laboratory, this evaluative process can be quantified by asking participants to rate how certain they are about their perceptual decisions, yielding what are commonly referred to as confidence judgments [5, 6, 7, 8].

An influential framework to model confidence judgments is Type II Signal Detection Theory (SDT) [9, 10], which provides a principled distinction between first‐order (perceptual) and second‐order (metacognitive) decisions [9, 10, 11]. Importantly, both levels are assumed to rely on a common computational logic: distinguishing signal from noise. At the perceptual level, Type I sensitivity (*d'*) indexes the ability to discriminate between relevant sensory signals and background noise, while the Type I bias (*criterion*) reflects the tendency to report “present” or “absent” responses, independently of Type I sensitivity. At the metacognitive level, Type II sensitivity (i.e., metacognitive sensitivity; *meta‐d*′) reflects the ability to discriminate between correct and incorrect first‐order responses, whereas Type II bias (i.e., metacognitive bias; *meta‐criterion*) captures a general tendency toward reporting higher or lower confidence, independently of Type II sensitivity.

Prior research has extensively investigated the neural underpinnings of confidence judgments [12, 13, 14, 15, 16, 17, 18, 19] and the role of prior expectations in shaping perceptual decisions [20, 21, 22, 23, 24, 25, 26, 27]. However, the neuro‐computational mechanisms through which prior expectations shape confidence judgments have received far less attention [28, 29, 30]. Within the Type II SDT framework, a key question is whether prior expectations bias confidence judgments (i.e., inducing a metacognitive bias) or instead modulate their accuracy (i.e., altering metacognitive sensitivity). This distinction carries important theoretical weight: evidence of a systematic metacognitive bias would indicate that confidence does not operate as an unbiased monitor of performance but is itself sensitive to prior information. Rather than representing a metacognitive failure, such susceptibility may reflect a self‐consistency mechanism that, under noisy sensory conditions, could help stabilize performance monitoring by anchoring confidence judgments to contextual priors. Building on these considerations and the established effect of prior expectations at the perceptual level—whereby expectations shift the Type I criterion without altering sensitivity [20, 22, 23, 24, 25, 31, 32]—we hypothesised that prior expectations would likewise shape confidence judgments, inducing a metacognitive bias while leaving metacognitive sensitivity unaffected.

At the neural level, alpha oscillations constitute a candidate mechanism supporting this integrative process. Recent electrophysiological studies have demonstrated that alpha‐band power is associated with confidence, as indicated by negative correlations between spontaneous pre‐stimulus alpha amplitude and subjective confidence [33, 34]. Crucially, expectation‐induced biases in Type I criterion have also been linked to modulations of pre‐stimulus alpha amplitude in parieto‐occipital regions [22, 23]. However, although these findings establish that (a) alpha activity correlates with confidence judgments and (b) prior expectations modulate alpha amplitude, it remains unclear whether these two lines of evidence converge at the Type II level—that is, whether cue‐induced modulation of alpha activity plays a causal role in integrating prior knowledge into confidence judgments.

To address whether (1) prior expectations influence confidence judgments and (2) cue‐induced pre‐stimulus alpha power modulation mediates this process, we conducted a first experiment combining electroencephalographic (EEG) recording with a behavioral task. Specifically, participants completed a visual detection task in which a probabilistic cue indicated the likelihood of the upcoming target. They were asked to report the presence or absence of the target (Type I response) and to rate their confidence in that decision (Type II response) on a 4‐point scale. We hypothesised (a) that prior information selectively shifts metacognitive bias without altering metacognitive sensitivity and (b) that cue‐induced modulation of pre‐stimulus alpha‐band amplitude is directly associated with shifts in metacognitive bias, with larger cue‐induced alpha modulation predicting a greater bias.

Regarding the cortical regions potentially involved in this process, converging evidence has indicated that parietal cortex (PC) activity is involved in the integration of prior knowledge [35, 36, 37], decision‐making under uncertainty [38], and metacognitive processes [39, 40]. Crucially, seminal studies have linked PC engagement to the regulation of alpha‐band rhythms [41, 42]. For instance, Capotosto and colleagues [43] demonstrated that inhibitory stimulation of PC disrupted alpha‐band dynamics, consistent with a role of the PC in shaping cortical excitability. This convergence makes PC a principled target for testing the causal role of alpha‐mediated prior integration into confidence judgments in perceptual tasks.

Building upon these findings and informed by the results from Study 1, we conducted a second experiment combining EEG and continuous Theta Burst Stimulation (cTBS) to test the causal involvement of PC in integrating prior expectations into confidence judgments. We hypothesised that inhibitory PC stimulation would temporarily disrupt participants' ability to modulate pre‐stimulus alpha‐band amplitude according to probabilistic cues. Consequently, this disruption should reduce the cue‐induced integration of prior information into confidence judgments, thereby diminishing the associated metacognitive bias. Importantly, this effect was expected to be specific to metacognitive bias, with no change in metacognitive sensitivity.

## 2 Results

### 2.1 Study 1

**FIGURE 1:**
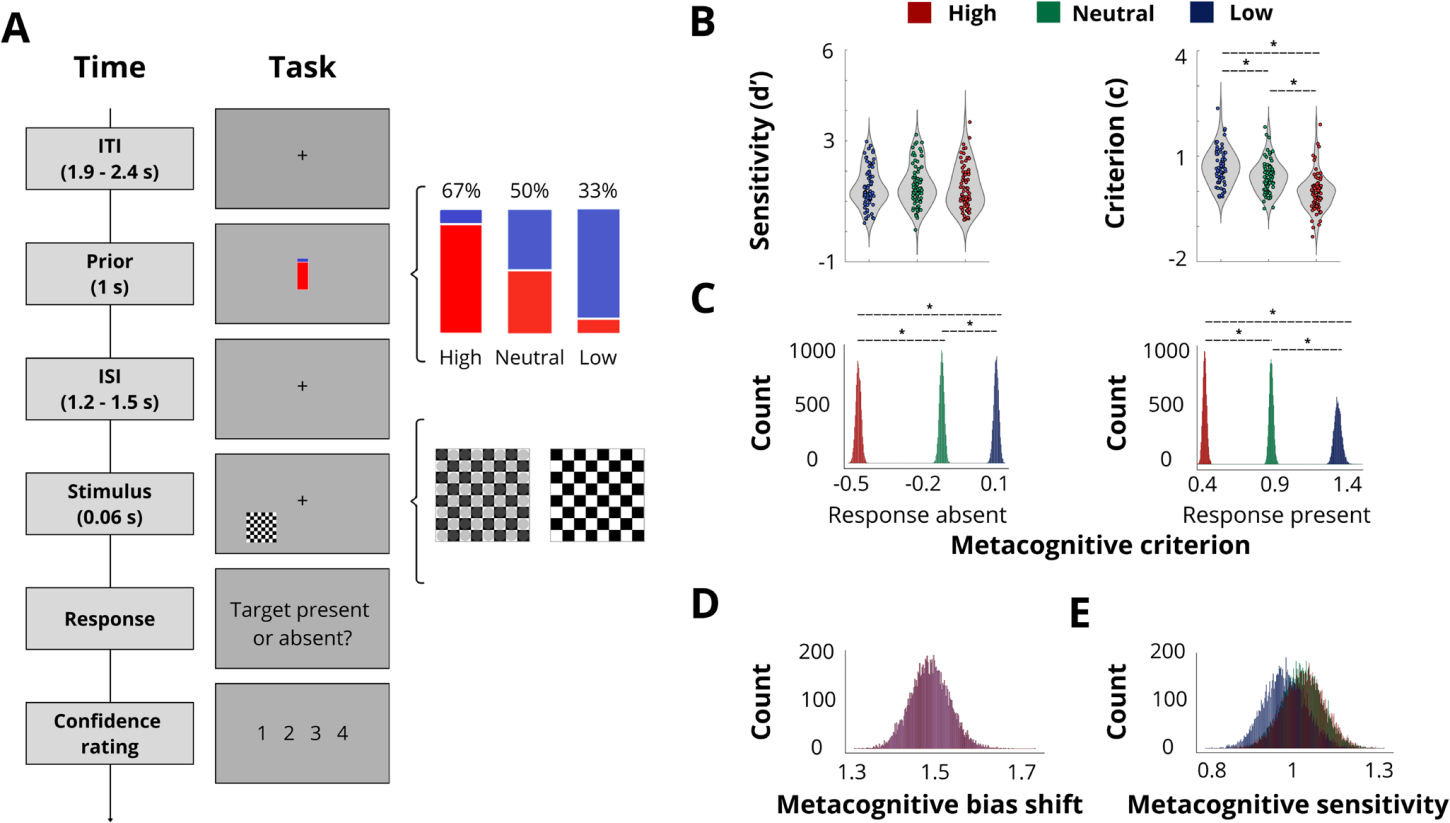
Experimental task and Study 1 results. (A) Experimental design. EEG data were collected during a simple visual detection task (participant = 75). Each trial began with a fixation cross, followed by the appearance of a probabilistic cue at the center of the screen. Subsequently, a checkerboard containing (or not) gray circles at the titrated contrast within it appeared in the bottom‐left of the screen for 60 ms. At the end of each trial, participants were asked to indicate how confident they were about their response. Confidence was rated using the keyboard keys “1”, “2”, “3”, and “4” corresponding, respectively to four levels of confidence: not confident at all (1), slightly confident (2), moderately confident (3), and highly confident (4). The cue was a rectangle with its bottom colored red and its top colored in blue. The percentage of the red shading to the entire rectangle indicated the target probability. Three cue levels were used: the high and low probability cues indicated the probability of the target presence of 67% and 33%, respectively, while the neutral cue predicted the target presence and absence equally (50%). (B) Type I Signal Detection Theory. Type I sensitivity (d′) and Type I bias (criterion) indices are represented separately for trials preceded by the high‐ (in red) low‐ (in blue), or neutral‐probability (in green) cues. The Y‐axis represents the values of the two parameters, and each circle corresponds to a subject. Asterisks correspond to significant differences. A repeated‐measure ANOVA showed that prior information did not affect Type I sensitivity. In contrast, the probabilistic cue significantly influenced the Type I bias: a more liberal criterion was adopted in trials preceded by the high probability cue, compared to both neutral and low probability cue. Conversely, a more conservative criterion was adopted in trials preceded by the low probability cue relative to the neutral cue (all *p* < 0.01). (C) Meta‐criterion. meta‐criterion index is represented separately for trials preceded by the high‐, low‐, or neutral‐probability cues for Type I responses (absent, present). The Y‐axis represents the count of observations across the HMeta‐d′ distribution, the X‐axis represents the most probable HMeta‐d′ values. Asterisks correspond to credible differences. A meta‐criterion value close to 0 indicates a more liberal criterion (i.e., tendency to give high‐confidence ratings), whereas a meta‐criterion value farther from 0 indicates a more conservative criterion (i.e., tendency to give low‐confidence ratings). The 95% Highest Density Interval (HDI) was computed for each cue. The group‐level posterior densities were then used to test the statistical significance of differences in meta‐criterion. When a Type I “present” response was given in trials preceded by a high‐probability cue, participants adopted a more liberal meta‐criterion (mean = 0.44) relative to trials preceded by neutral‐probability cue (mean = 0.84; HDI_high‐neutral_ [–0.45, –0.34]) and low‐probability cue (mean = 1.25; HDI_high‐low_ [–0.87, –0.75]) in which participants adopted a more conservative meta‐criterion relative to the neutral condition (HDI_low‐neutral_ [0.35, 0.47]). Conversely, when a Type I “absent” response was given in trials preceded by a high‐probability cue, participants adopted a more conservative meta‐criterion (mean = –0.54) compared to trials preceded by a neutral‐probability cue (mean = –0.13; HDI_high‐neutral_ [–0.45, –0.36]), and low‐probability cue (mean = 0.14; HDI_high‐low_ [–0.73, –0.63]) in which participants adopted a more liberal meta‐criterion relative to the neutral condition (HDI_low‐neutral_ [0.23, 0.32]). (D) Metacognitive bias shift. Higher values indicate greater reliance on probabilistic information when making confidence judgments (see Methods). (E) Metacognitive sensitivity (meta‐d′). meta‐d′ index is represented separately for trials preceded by the high‐, low, or neutral‐probability cues. Higher values correspond to greater metacognitive ability. The 95% Highest Density Interval (HDI) was computed for each cue. The group‐level posterior densities were then used to test the statistical significance of differences in metacognitive sensitivity. Prior information did not affect metacognitive sensitivity. The analysis revealed that the probabilistic cue did not affect meta‐d′ (figure 1E; mean low‐probability = 1.08; neutral‐probability = 1.07; high‐probability = 1.01; HDI_low‐high_ [–0.04, 0.19]; HDI_neutral‐high_ [–0.07, 0.17]; HDI_neutral‐low_ [–0.14, 0.09]).

Seventy‐five participants completed a basic visual detection task (Figure 1A). On each trial, a checkerboard appeared in the lower‐left visual field. The checkerboards either included isoluminant gray circles within their cells (target trials) or were empty (catch trials). Participants were instructed to use the keyboard to indicate whether the target was present, followed by a confidence judgment on their performance. Confidence was rated using the keyboard keys “1”, “2”, “3”, and “4” corresponding, respectively to four levels of confidence: not confident at all (1), slightly confident (2), moderately confident (3), and highly confident (4). A symbolic cue preceded each checkerboard, indicating the probability of target presence. Three types of cues were used: a high‐probability cue (67%), a low‐probability cue (33%), and a neutral‐probability cue (50%) indicating equal probability of presence and absence. The actual presence of the targets matched the probabilities signaled by the cues, and participants were explicitly informed about that.

#### 2.1.1 Prior Information Shapes Decision‐Making Strategies

To investigate the effect of the expectancy cue on perceptual decision‐making, the Type I SDT indices *d*′ and *criterion* were calculated separately (Figure 1B). The analysis showed that cues affected Type I bias (F_2,148_ = 84.49; *p* < 0.01; η_p_^2^ = 0.53) but not Type I sensitivity (F_2,148_ = 1.38; *p* > 0.25; η_p_^2^ = 0.02). Specifically, the participants adopted a more liberal *criterion* in trials preceded by the high‐probability cue (mean ± SEM = −0.02 ± 0.06) relative to trials preceded by the neutral‐probability cue (0.42 ± 0.05; t_74_ = −9.15; *p* < 0.01) and low‐probability cue (0.69 ± 0.06; *t*_74_ = −9.72; *p* < 0.01), in which participants adopted a more conservative *criterion* relative to the neutral condition (t_74_ = 7.02; *p* < 0.01). Overall, the cue selectively manipulates Type I bias without affecting Type I sensitivity.

#### 2.1.2 Prior Information Shapes Metacognitive Bias but Not Metacognitive Sensitivity

To investigate the effect of the expectancy cue on confidence judgments, the Type II SDT indices *meta‐dʹ* and *meta‐criterion* were calculated separately for trials preceded by the high‐, low‐, and neutral‐probability cues using *HMeta‐d*′ toolbox [44]. The analysis showed that prior probabilistic information shaped *meta‐criterion* (Figure 1C). Specifically, when a Type I “present” response was given in trials preceded by a high‐probability cue, participants adopted a more liberal *meta‐criterion* (mean = 0.44) relative to trials preceded by the neutral‐probability cue (mean = 0.84; HDI_high‐neutral_ [–0.45, –0.34]) and low‐probability cue (mean = 1.25; HDI_high‐low_ [–0.87, –0.75]) in which participants adopted a more conservative *meta‐criterion* relative to the neutral condition (HDI_low‐neutral_ [0.35, 0.47]). Conversely, when a Type I “absent” response was given in trials preceded by a high‐probability cue, participants adopted a more conservative *meta‐criterion* (mean = –0.54) compared to trials preceded by a neutral‐probability cue (mean = –0.13.; HDI_high‐neutral_ [–0.45, –0.36]), and low‐probability cue (mean = 0.14; HDI_high‐low_ [–0.73, –0.63]) in which participants adopted a more liberal *meta‐criterion* relative to the neutral condition (HDI_low‐neutral_ [0.23, 0.32]). Furthermore, the analysis revealed that the probabilistic cue did not affect *meta‐d*′ (Figure 1E; mean low‐probability = 1.08; neutral‐probability = 1.07; high‐probability = 1.01; HDI_low‐high_ [–0.04, 0.19], mean_low‐high_ = 0.07; HDI_neutral‐high_ [–0.07, 0.17], mean_neutral‐high_ = 0.05; HDI_neutral‐low_ [–0.14, 0.09], mean_neutral‐low_ = –0.02). These findings suggest that the prior information conveyed by the cue does not modulate participants′ metacognitive sensitivity but rather induces a metacognitive bias in evaluating the correctness of their choice. Specifically, this effect was congruence‐dependent: the same cue modulated metacognitive bias differently depending on whether the participant's perceptual response was congruent or incongruent with it. Finally, a global *metacognitive bias shift* was calculated (see Methods) (Figure 1D), with an average value of 1.50 (HDI [1.43, 1.56]). Increasing values in this metric reflect an enhanced weighting of probabilistic information in the formation of confidence judgments. A control analysis using the m‐distance metric [45] confirmed that this pattern is preserved after controlling for Type I *criterion shift* (see Figure S2).

#### 2.1.3 Pre‐Stimulus Alpha Dynamics Encode Expectancy Information

**FIGURE 2:**
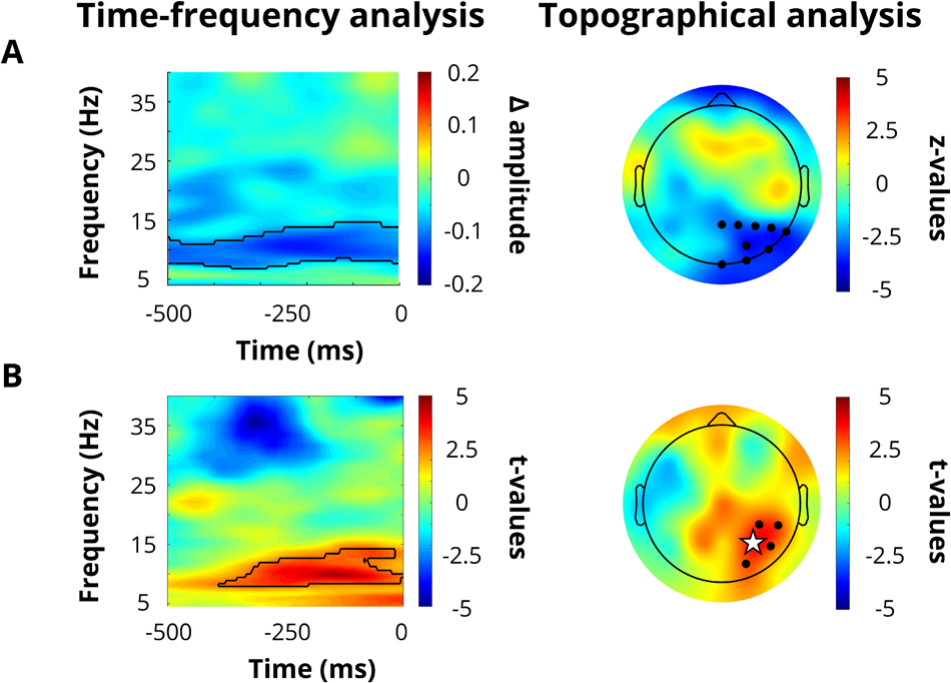
Parietal alpha dynamics predict metacognitive bias shift. (A) (left). Time–frequency map of the pre‐stimulus (−500–0 ms; X‐axis) amplitude difference between trials preceded by the high‐ and low‐probability cue (Δ amplitude), recorded in posterior electrodes (Participant = 75). Time 0 corresponds to stimulus onset. A cluster permutation analysis was performed. Black contours indicate statistically significant clusters. The Y‐axis represents the frequency (4–40Hz). There is a suppression in pre‐stimulus alpha amplitude in high‐probability trials compared to low‐probability trials (*p* < 0.01). A (right). Topography of the differential activations in the alpha‐band (∼7–14 Hz) between trials preceded by the high‐ and low‐probability cue during the pre‐stimulus window (−500–0 ms). Black dots indicate statistically significant electrodes (Pz, P2, P4, P6, P8, PO4, PO8, O2, Oz). The colorbar represents z‐values of Wilcoxon signed‐rank test between high‐probability cue and low‐probability cue. Alpha‐band amplitude diverges in high‐ compared to low‐probability trials over posterior electrodes in the right hemisphere. (B) (left) The results of a brain‐to‐behavioral analysis on amplitude difference between the high‐ and low‐probability cues across a range of times (−500–0) and frequencies (4–40 Hz). Time 0 corresponds to stimulus onset. Black contours indicate statistically significant clusters. There is a positive correlation in a cluster centered within the alpha band: the greater the cue‐induced modulation of alpha‐band amplitude, the stronger the induced metacognitive bias shift. B (right). Topography of the significant electrodes in the brain‐to‐behavior analysis conducted between Δ alpha amplitude (∼7–14 Hz) extracted from each electrode (−500–0 ms) and metacognitive bias shift. The analysis revealed a cluster of electrodes located in the right parieto‐occipital region (PO4, P4, P6, CP4, CP6) that showed a strong association with metacognitive bias shift, with electrode P4 (star) exhibiting the most robust effect (t value = 3.83, *p* < 0.001).

To examine whether pre‐stimulus electroencephalographic activity over posterior electrodes was influenced by the probabilistic information provided by the cue, a non‐parametric cluster‐based permutation test across the time × frequency domain was conducted (Figure 2A). Specifically, the spectral amplitude of trials preceded by a high‐probability cue against those preceded by a low‐probability cue was contrasted, focusing on the 500‐ms interval before stimulus onset and covering a broad frequency range from 4 to 40 Hz. This approach allowed identifying spatiotemporally contiguous regions of significant difference without assuming parametric distributions. The analysis revealed a reliable modulation in the alpha band, such that high‐probability cues were associated with a stronger suppression of pre‐stimulus posterior alpha amplitude compared to low‐probability cues (*p* < 0.01). This finding indicates that probabilistic expectations selectively influenced anticipatory neural dynamics prior to sensory processing. Subsequently, a topographical analysis was performed to examine the spatial distribution of the observed pre‐stimulus effects (Figure 2A). This involved contrasting high‐ versus low‐probability cue trials for each electrode in the alpha frequency range (∼7–14 Hz) within a 500‐ms pre‐stimulus interval. The topographic cluster‐based permutation test revealed a spatially specific modulation of alpha‐band amplitude over right parieto‐occipital electrodes. A complementary 3D cluster‐based permutation test (time × frequency × electrode) confirmed the same pattern of results (see Figure S3).

#### 2.1.4 Pre‐Stimulus Alpha Band Modulation Affects Metacognitive Bias Shift

To assess the relationship between the behavioral modulations induced by the expectancy cue and the corresponding oscillatory dynamics, a correlation analysis was performed between participants′ *metacognitive bias shift* and the trial‐type amplitude difference (high‐ vs. low‐probability cue) at each time–frequency point within the 500‐ms pre‐stimulus window, spanning the 4–40 Hz frequency range (Figure 2B). This approach allowed directly linking individual differences in behavior with neural oscillatory activity, identifying regions in the time–frequency space where variability in oscillatory dynamics stemming from the provided prior systematically covaried with the magnitude of the *metacognitive bias shift*. The analysis revealed a significant cluster centered within the alpha band (*p* < 0.01), indicating that pre‐stimulus, cue‐induced alpha modulation is functionally tied to the observed behavioral effect of probabilistic expectations. Specifically, a stronger prior‐driven modulation of alpha amplitude corresponds to a greater *metacognitive bias shift*. To further investigate this relationship at a topographical level, a correlation analysis was conducted between Δ alpha amplitude (∼7–14 Hz) extracted from each electrode (time: –500–0 ms) and the *metacognitive bias shift* (Figure 2B). The analysis revealed a cluster of electrodes located in the right parietal region showing a prominent association with *metacognitive bias shift*. This confirms previous studies that showed a robust relationship between parietal activity and metacognitive processes [39, 46, 47]. To inform the stimulation target for Study 2, we identified the specific electrode within this cluster that exhibited the strongest brain–behavior correlation, with P4 (represented by a star in Figure 2B) showing the most robust association (t value = 3.83, *p* < 0.001).

### 2.2 Study 2

**FIGURE 3:**
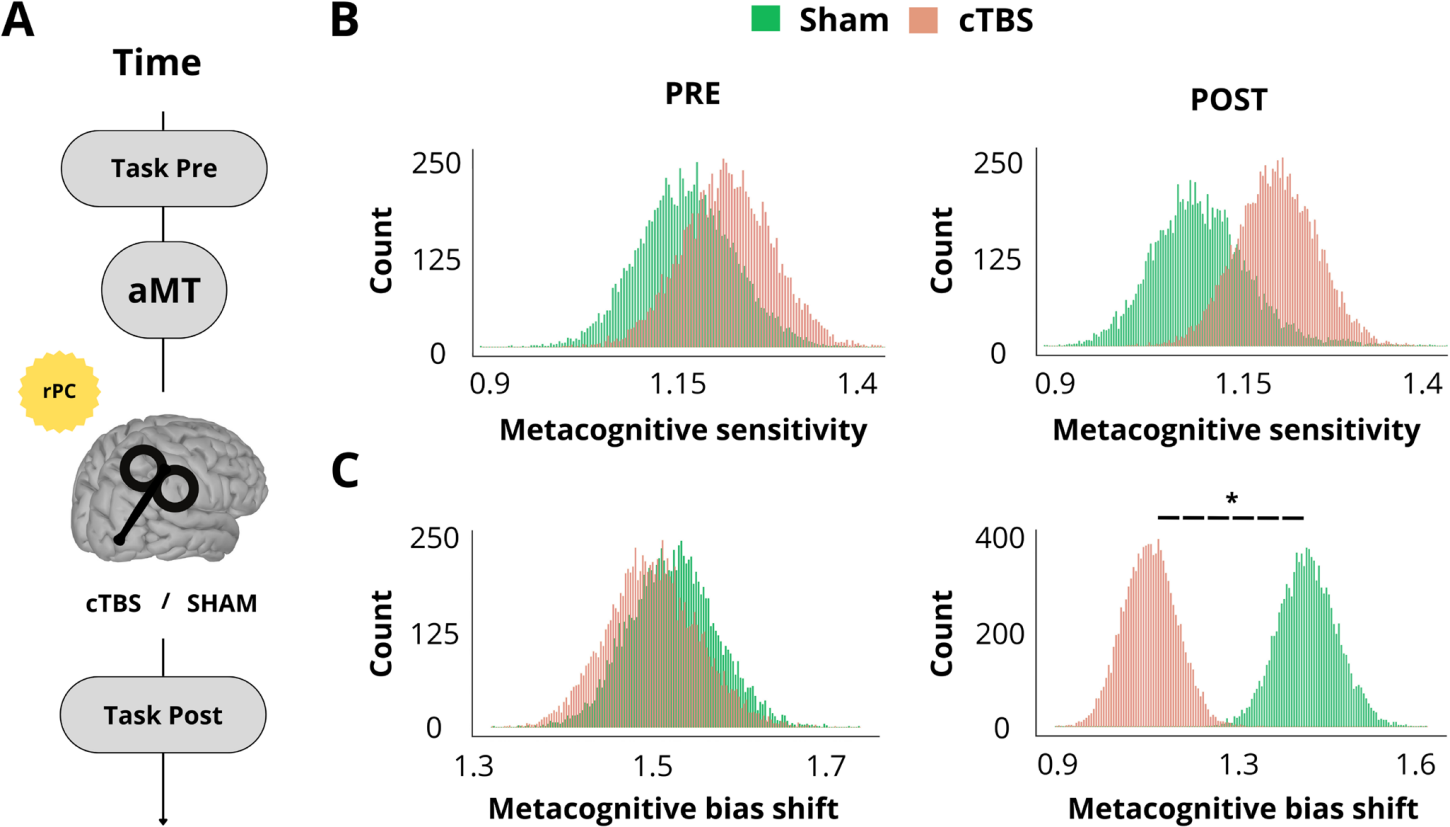
Parietal cTBS selectively reduced metacognitive bias shift without affecting metacognitive sensitivity. (A) Experimental design. EEG data were collected during the same visual detection task used in Study 1. Participants (n = 88) completed three experimental blocks, after which the active motor threshold (aMT) was determined to set the stimulation intensity of the TMS protocol. Then, the continuous Theta Burst Stimulation (cTBS) or SHAM stimulation protocol was applied (N = 44 in each group) over the right parietal cortex. After the stimulation, participants performed three experimental blocks again to test the TMS‐related effect on confidence judgments. (B) Metacognitive sensitivity (meta‐d′). meta‐d′ index is represented in PRE and POST sessions for both SHAM and cTBS conditions. Values represent the group‐level posterior distribution from the hierarchical model. The Y‐axis represents the count of observations across the HMeta‐d′ distribution, and the X‐axis represents the most probable HMeta‐d′ values. Higher Meta‐d′ values indicate greater metacognitive ability. No significant changes in meta‐d' were observed following stimulation, indicating that neither cTBS nor SHAM affected metacognitive sensitivity. (C) Metacognitive bias shift. Asterisks correspond to credible differences. The group‐level posterior densities were then used to test the statistical significance of differences in metacognitive bias shifts between the two groups. Higher values indicate greater reliance on probabilistic information when making confidence judgments. The index is represented in PRE and POST sessions for both SHAM and cTBS conditions. cTBS led to a credible reduction in metacognitive bias shift compared to SHAM stimulation.

Study 1 revealed that the expectancy cue modulates confidence judgments by inducing a metacognitive bias without altering metacognitive sensitivity. Moreover, this behavioral effect was associated with cue‐induced alpha modulation recorded from parietal electrodes, which tracked prior‐driven changes in confidence judgments. Therefore, a second study was conducted in which 88 participants completed the same task as in Study 1, with forty‐four receiving continuous Theta Burst Stimulation (cTBS) and forty‐four receiving SHAM stimulation. This design allowed us to test the causal role of the PC in shaping metacognitive bias following the induction of prior expectations (Figure 3A).

#### 2.2.1 cTBS Does Not Affect Type I Decision

The Type I SDT indices *d*′ and *criterion* were calculated to compare the effect of the expectancy cue before (PRE) and after (POST) the two types of stimulation (cTBS, SHAM; see “Supporting Information”). Consistent with the result of Study 1, the ANOVA revealed that the cues modulated the Type I bias (F_2,88_ = 81.99; *p* < .01; η_p_^2^ = 0.49) but not Type I sensitivity (F_2,88_ = 1.74; *p* = 0.18; η_p_^2^ = 0.02). Specifically, post‐hoc analysis showed that in trials where the high‐probability cue was presented, participants adopted a more liberal *criterion* (mean ± SEM = −0.09 ± 0.06) compared to trials with the neutral‐probability cue (0.29 ± 0.05; *t*_86_ = 8.20; *p* < 0.01) and the low‐probability cue (0.63 ± 0.05; *t*_86_ = 9.56; p < 0.01), in which participants adopted a more conservative *criterion* relative to the neutral condition (*t*_86_ = 8.29; *p* < 0.01). Crucially, no main effects of stimulation or session were found for either *d*′ or *criterion*, nor were there any significant interactions between stimulation, sessions, and cue (all Fs< 2.02; all *p* > 0.14). This result indicates that the type of cue (high‐, low‐, or neutral‐probability) modulates the Type I bias but not Type I sensitivity, independently of stimulation type (cTBS, SHAM) and sessions (PRE, POST). Overall, the behavioral results indicate that inhibitory stimulation did not affect either the effect of the cue on Type I bias or Type I sensitivity.

#### 2.2.2 Disrupting Parietal Cortex Activity Abolished Cue Effects on Metacognitive Bias Shift

The Type II SDT indices *meta‐d*′ and *metacognitive bias shift* were calculated before (PRE) and after (POST) the two types of stimulation (cTBS, SHAM) to investigate whether the effect of the expectancy cues depends on the type of stimulation received by the participants. The analysis showed a credible reduction in *metacognitive bias shift* from PRE to POST in the cTBS group (mean PRE = 1.56; mean POST = 1.16; mean PRE—mean POST = 0.41; HDI [0.27, 0.55]). In the SHAM group, the corresponding PRE to POST change was smaller in magnitude (mean PRE = 1.60; mean POST = 1.44; mean PRE—mean POST = 0.16; HDI [0.02, 0.29]). Critically, a credible difference was present in the POST session (mean cTBS—mean SHAM = –0.27; HDI [–0.41, –0.14]), showing that *metacognitive bias shift* was substantially lower after cTBS stimulation relative to SHAM, while the two groups were comparable at baseline, as no credible difference emerged in the PRE session (mean cTBS—mean SHAM = –0.03; HDI [–0.17, 0.11]). This pattern was directly supported by the Stimulation × Session interaction computed on the posterior distribution of the *metacognitive bias shift*, which was credible (HDI [0.04, 0.44]). This analysis indicates that, while the two groups showed comparable levels of prior‐related bias in confidence at PRE, this pattern diverged following stimulation, with the cTBS group exhibiting a larger reduction in *metacognitive bias shift* than the SHAM group at POST (Figure 3C).

#### 2.2.3 Metacognitive Sensitivity and Raw Confidence Levels Are Unaffected by Parietal Stimulation

Because Study 1 showed that probabilistic cues did not modulate *meta‐d'*, the cue factor was collapsed for the analysis of metacognitive sensitivity. *meta‐d'* did not differ significantly in either the PRE (mean cTBS = 1.23; mean SHAM = 1.19; mean cTBS—mean SHAM = 0.03; HDI [–0.12, 0.19]) or POST sessions (mean cTBS = 1.17; mean SHAM = 1.04; mean cTBS—mean SHAM = 0.13; HDI [–0.03, 0.29]). Within‐group comparison between the PRE and POST sessions further confirms the absence of differences (mean cTBS = 0.06; HDI [–0.10, 0.22]; mean SHAM = 0.16; HDI [–0.01, 0.32]). This implies that the type of stimulation delivered did not induce changes in the participants′ metacognitive sensitivity (Figure 3B). A control analysis on mean raw confidence ratings confirmed that cTBS did not induce a global shift in confidence level, indicating that stimulation selectively targeted the integration of prior information into confidence judgments (see Supporting Information). Overall, this evidence shows that inhibitory stimulation of the parietal area, but not SHAM stimulation, reduced the use of expectation‐like information in confidence judgments, thereby reducing the metacognitive bias shift.

#### 2.2.4 cTBS Affects Pre‐Stimulus Prior Induced Alpha Modulation

**FIGURE 4:**
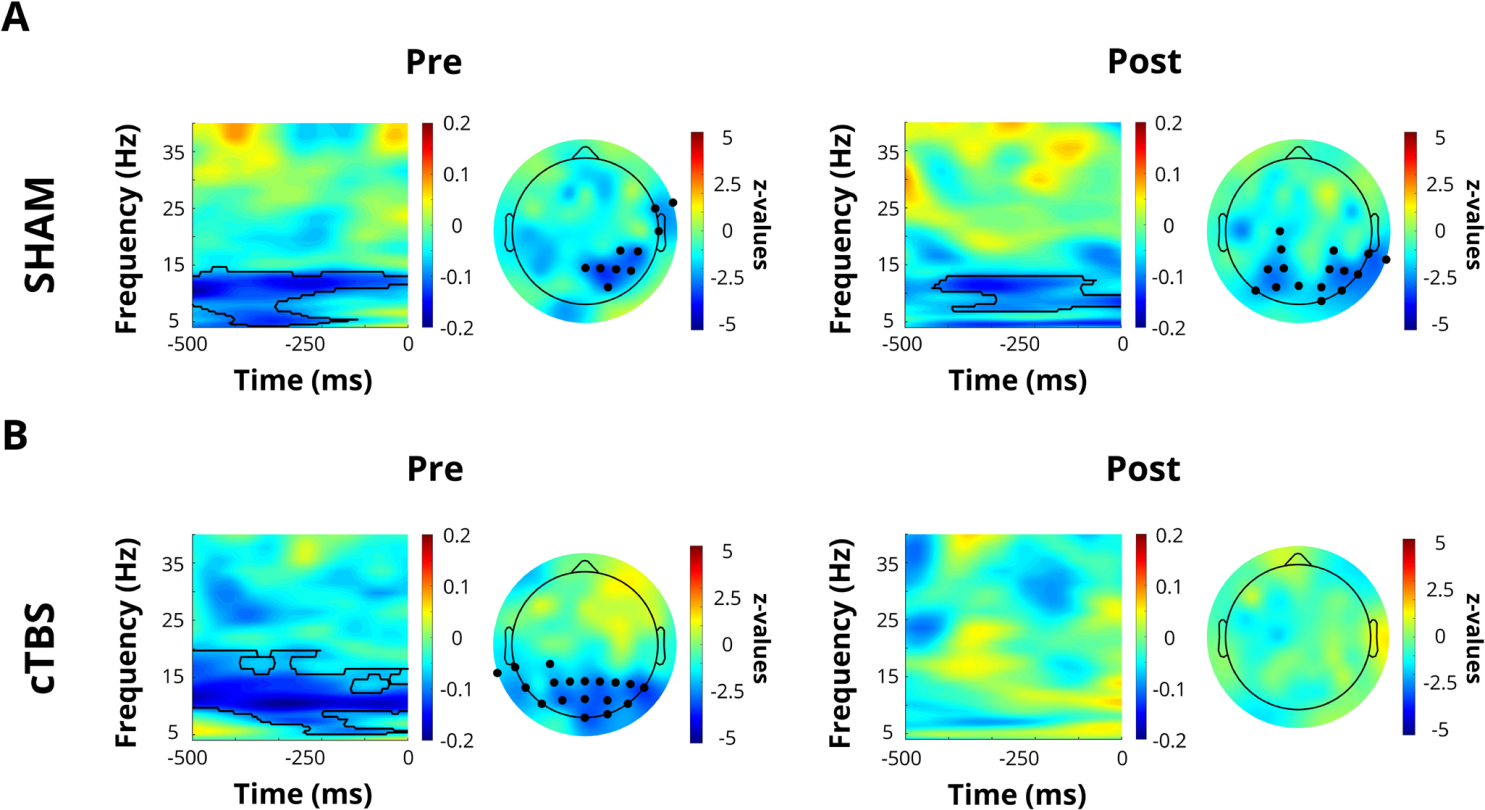
Parietal cTBS abolishes cue‐induced alpha amplitude modulation. (A) Time–frequency representations of amplitude differences between high‐ and low‐probability cue trials at right posterior electrodes, for the SHAM group (participant = 44). A cluster permutation analysis was performed. In both PRE and POST sessions, a significant cluster centered within the alpha band was found (*p* < 0.05). Corresponding scalp topographies computed over the alpha‐band cluster reveal that the effect is spatially specific to posterior sites. In both PRE and POST sessions, a significant cluster of modulation centered within the alpha‐band emerged (*p* < 0.05; PRE: Pz, P2, P4, P6, CP4, CP6, T8, FC8, FT10; POST: O2, PO8, PO4, PO7, PO3, POz, P4, P8, P6, CP4, CP8, TP10, P1, P3, CP1, C1). (B) Time–frequency representations of pre‐stimulus amplitude differences between high‐ and low‐probability cue trials at posterior electrodes, for the cTBS group (participant = 44). A cluster permutation analysis was performed. In the PRE session, a significant pre‐stimulus cluster of modulation emerges (*p* < 0.05). The topography computed confirms spatial specificity to posterior sites (*p* < 0.05). (PRE: Oz, O2, PO8, POz, PO4, PO3, PO7, Pz, P2, P4, P6, P8, P1, P3, P7, CP3, TP7, TP9). By contrast, in the POST session no significant cluster is observed and the topography map shows no electrodes reaching significance, indicating that parietal cTBS abolished the cue‐induced modulation of the pre‐stimulus alpha rhythm.

To assess the effect of cTBS on cue‐based regulation of pre‐stimulus oscillatory activity on posterior electrodes, we conducted a non‐parametric cluster‐based permutation test across the time × frequency domain in PRE and POST sessions separately for cTBS and SHAM groups (Figure 4A,B). Specifically, we contrasted the spectral amplitude of trials preceded by a high‐probability cue against those preceded by a low‐probability cue, focusing on the 500‐ms interval before stimulus onset and covering a broad frequency range from 4 to 40 Hz. The analysis of the PRE session showed that, in both stimulation conditions, there was a suppression of alpha amplitude in trials preceded by a high‐probability cue compared to those preceded by a low‐probability cue (*p* < 0.05), mimicking the results obtained in Study 1. Moreover, a pooled PRE analysis combining SHAM and cTBS participants (N = 88) confirmed that the brain–behavior association between cue‐induced alpha shift and *metacognitive bias shift*, observed in Study 1, is replicated in this independent sample (slope = 0.295, t = 3.185, p = 0.002; see Figure S6 for spectrotemporal and spatial characterization). The alpha shift × group interaction was not significant (Δslope = −0.170, *p* = 0.38), indicating that the brain–behavior relationship did not differ between SHAM and cTBS.

In the POST session, however, in the cTBS group no differences in alpha amplitude were observed between the two conditions (*p* = 0.88), suggesting that parietal cTBS stimulation prevented participants from modulating their alpha rhythm in an expectation‐driven fashion. The specificity of the stimulation is confirmed by the fact that, in the SHAM group, cue‐induced pre‐stimulus alpha amplitude modulation persisted (*p* < 0.05), suggesting that participants continued to suppress alpha rhythm after the presentation of high‐probability vs. low‐probability cues. Crucially, a direct Stimulation x Session interaction test confirmed that the PRE–POST reduction in cue‐induced alpha modulation was larger after cTBS than after SHAM (*p* = 0.046), supporting a stimulation‐specific attenuation of expectancy‐related alpha modulation (see Supporting Information). In addition, a control analysis on cue‐averaged alpha power (see Supporting Information) indicated that cTBS did not induce a global shift in pre‐stimulus alpha activity, suggesting that the stimulation effect was specific to cue‐induced alpha modulation rather than a general shift in alpha amplitude.

Subsequently, a topographical analysis was performed to examine the spatial distribution of the observed pre‐stimulus effects in PRE and POST sessions separately for cTBS and SHAM groups (Figure 4A,B). This involved contrasting high‐ versus low‐probability cue trials for each electrode in an alpha frequency range (∼7–14 Hz) focusing on a 500‐ms pre‐stimulus interval. In the PRE session, the topographic cluster‐based permutation test revealed a spatially specific modulation of cue‐induced alpha activity over parieto‐occipital electrodes in both the SHAM and cTBS groups. In the POST session, the SHAM group continued to show a cue‐induced modulation of alpha activity over parieto‐occipital electrodes, whereas no significant cluster emerged in the cTBS group. These results confirm, at a topographical level, that inhibitory parietal stimulation prevents participants from modulating their alpha rhythm in an expectation‐driven fashion. A complementary 3D cluster‐based permutation test (time × frequency × electrode) confirmed the same pattern of results (see Figure S3).

#### 2.2.5 cTBS‐Induced Reduction in Metacognitive Bias Shift is Mediated by Changes in Cue‐Induced Alpha Modulation

**FIGURE 5:**
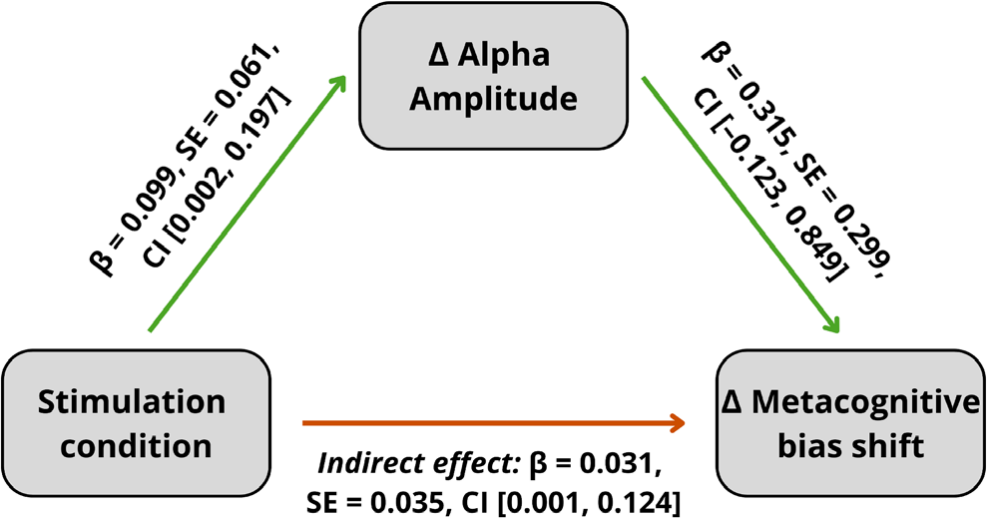
Inhibiting PC activity reduces metacognitive bias shift via attenuation of cue‐induced alpha modulation. A simple mediation model (PROCESS Model 4) tested whether the effect of stimulation condition (cTBS vs. SHAM; X) on the PRE‐to‐POST changes in metacognitive bias shift (Y) was transmitted via the PRE‐to‐POST changes in cue‐induced pre‐stimulus alpha modulation (M). Cue‐induced alpha modulation was first computed within each session as alpha shift = alpha amplitude_low‐probability cue − alpha amplitude_high‐probability cue. The mediator was then computed as Δ alpha amplitude = alpha shift_PRE − alpha shift_POST, such that positive values indicate a reduction in cue‐induced alpha modulation from PRE to POST. The outcome was computed analogously as Δ metacognitive bias shift = metacognitive bias shift_PRE − metacognitive bias shift_POST, such that positive values indicate a reduction in metacognitive bias shift after stimulation. cTBS (relative to SHAM) was associated with a larger PRE‐to‐POST reduction in cue‐induced alpha modulation (path a: β = 0.099, CI [0.002, 0.197]). The total (c: β = 0.243, CI [−0.012, 0.564]) and direct effects (c′: β = 0.212, CI [−0.053, 0.520]) were not statistically significant. Crucially, the indirect effect (a × b) was statistically significant (β = 0.031; CI [0.001, 0.124]), consistent with indirect‐only mediation in which cue‐driven modulation of alpha amplitude statistically mediates the relationship between parietal cTBS and changes in metacognitive bias shift.

Finally, a mediation analysis (Figure 5) was conducted to test the hypothesis that changes in cue‐induced alpha amplitude modulation statistically mediated the relationship between stimulation conditions (cTBS vs. SHAM) and the modulation of *metacognitive bias shift*. The mediation pathway showed significant components: cTBS, relative to SHAM, was associated with a larger PRE‐to‐POST reduction in cue‐induced alpha modulation (a = 0.099, SE = 0.061, CI [0.002, 0.197]). Critically, this reduction in alpha modulation statistically mediated the PRE‐to‐POST reduction in metacognitive bias shift, as indicated by a significant indirect effect (indirect effect = 0.031, SE = 0.035, CI [0.001, 0.124]). Neither the total effect (c = 0.243, SE = 0.173, 95% CI [–0.012, 0.564]) nor the direct effect (c′ = 0.212, SE = 0.174, 95% CI [–0.053, 0.520]) reached significance. This pattern, characterized by a significant indirect effect in the absence of significant total or direct effects, is consistent with indirect‐only mediation [48], indicating that PRE‐to‐POST alpha amplitude modulation accounts for the stimulation‐related change in *metacognitive bias shift*.

## 3 Discussion

Metacognition lies at the core of adaptive behavior, allowing individuals to regulate their actions by assessing confidence in their perceptual decisions. Here, in two complementary studies, we (1) characterized the electrophysiological dynamics by which prior knowledge biases confidence judgments and (2) established the causal contribution of the parietal cortex to this bias.

In the first study, we employed a probabilistic detection task in which symbolic cues conveyed the likelihood of target presence, combined with EEG recordings from a large sample of 75 participants. Behavioral data revealed that prior expectations systematically affected the Type I bias (*criterion*) rather than Type I sensitivity (*d*′): participants adopted a more liberal *criterion* when the cue signaled high stimulus probability, and a more conservative *criterion* when the cue signaled low probability, replicating established effects of prior expectations on Type I bias [20, 21, 22, 49, 50].

Importantly, we also observed that prior expectations induced a systematic metacognitive bias: participants expressed higher confidence when their perceptual decisions aligned with prior expectations and lower confidence when decisions contradicted them. This *metacognitive bias shift* occurred without any change in metacognitive sensitivity, indicating that expectations selectively shift confidence judgments rather than the ability to discriminate correct from incorrect decisions. This congruence‐dependent pattern suggests that, in our paradigm, confidence tracked perceptual self‐consistency: when sensory readout matched internal models, confidence increased; when the two diverged, confidence decreased, possibly signaling epistemic conflict between two different sources of information. One possible implication is that such a mechanism may support efficient decision‐making under uncertainty by reducing the need for continuous re‐evaluation [51]. However, the same mechanism may become maladaptive if confidence judgments become overly tied to internal coherence and less sensitive to disconfirmatory sensory evidence, progressively impairing metacognitive sensitivity. This interpretation is consistent with evidence showing that confidence can gate post‐decisional evidence accumulation [52], as well as with clinical and subclinical findings linking psychosis‐spectrum phenomena to overconfidence in errors, prior overweighting, belief inflexibility, and abnormalities in confidence discrimination [53, 54, 55, 56, 57, 58].

Building on previous research into how prior expectations shape decision‐making [21, 22, 23, 59, 60], our findings offer a new perspective: prior knowledge does not just shape perception or choice, it also biases how people evaluate their own decisions. Having established that prior expectations selectively affect metacognitive bias, we characterized the neural oscillatory dynamics underpinning this effect. EEG analyses revealed a robust expectation‐driven modulation of alpha‐band amplitude: high‐probability cues induced a significantly stronger pre‐stimulus alpha amplitude desynchronization than low‐probability cues [22]. Moreover, a direct brain–behavior correlation showed that the magnitude of this alpha‐band modulation predicted the degree of *metacognitive bias shift*, indicating that alpha desynchronization tracks reliance on prior beliefs during confidence judgments. While earlier studies established that spontaneous pre‐stimulus alpha amplitude predicts whether perceptual confidence will be high or low [33, 34], indexing fluctuations in cortical excitability [61] that shape perceptual certainty [62], our results uncover a functionally distinct mechanism. Specifically, we show that alpha activity is not merely linked to the level of confidence, but also to the extent to which confidence judgments are biased by high‐level information such as probabilistic prior expectations. This transition points to a broader functional role of alpha variability: rather than merely mirroring cortical excitability, it gates the way internal predictions shape subjective confidence in perceptual decisions.

**FIGURE 6:**
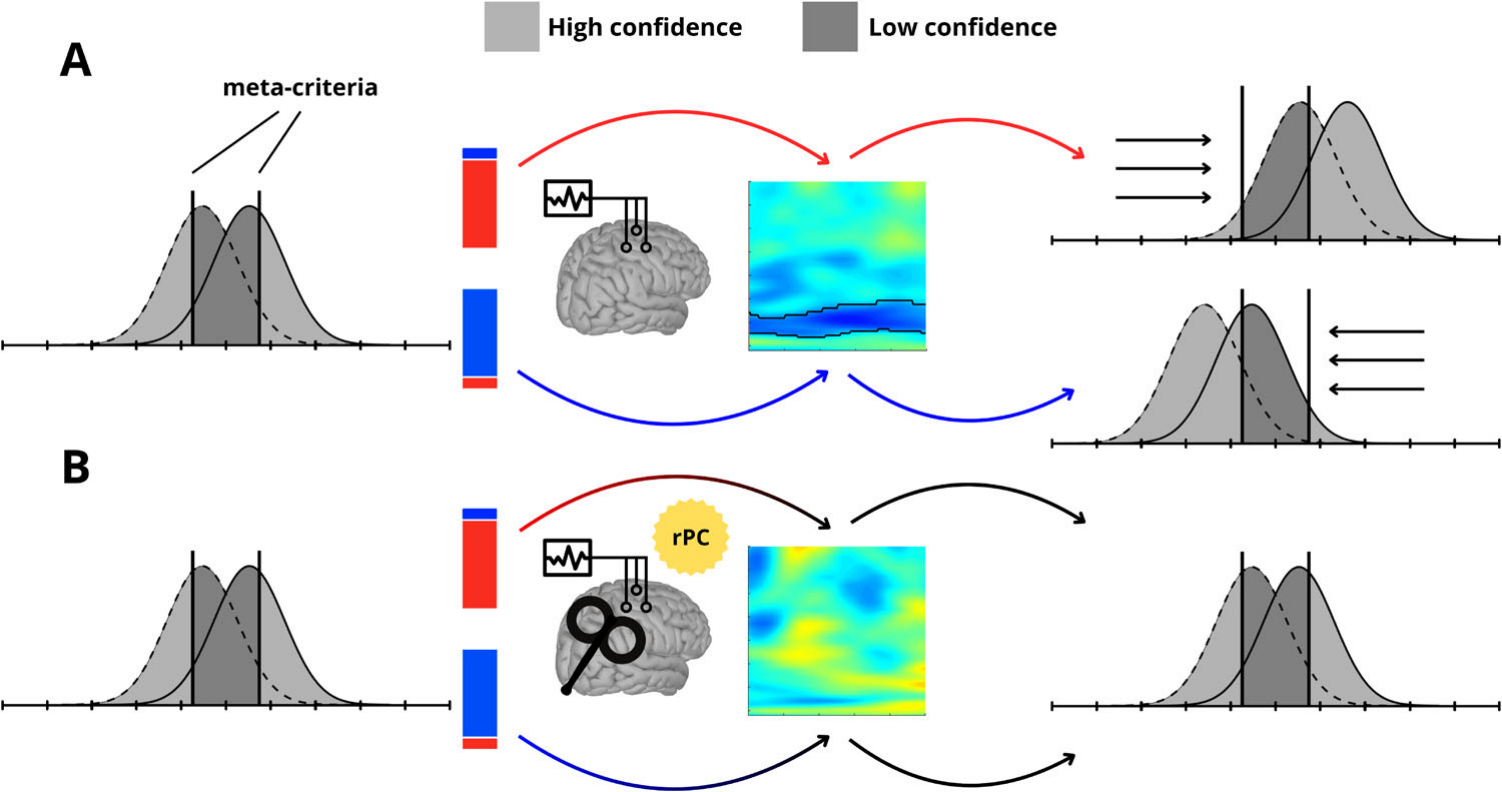
Alpha‐driven shifts in the internal response distributions bias confidence. (A) The figure represents a schematic account of how cue‐induced alpha rhythm modulation would induce a metacognitive bias. Within the SDT framework, a high‐confidence judgment is given when the internal response exceeds the meta‐criteria; otherwise, a low‐confidence judgment is provided. As indicated by the position of the “signal absent” (dashed line) and “signal present” (solid line) distributions relative to their own meta‐criteria, an unbiased observer has an equal probability of giving high‐ (light gray) or low‐ (dark gray) confidence judgments. When the probabilistic cue is presented, there is a modulation of the alpha rhythm dependent on the information conveyed by the cue. In turn, this modulation of alpha amplitude leads to a shift in the internal distributions while the meta‐criteria remain stable. In the figure, these dynamics are illustrated by the difference in alpha amplitude between the two cues. Specifically, desynchronization (after high‐probability cue) increases cortical excitability and, consequently, leads to a rightward shift in the internal response distribution. This shift results in a systematic bias that increases the likelihood of giving a high confidence “signal present” response and decreases the likelihood of giving a high‐confidence “signal absent” response. Conversely, alpha synchronization (after low‐probability cue) reduces cortical excitability and, consequently, leads to a leftward shift in the internal response distribution, increasing the likelihood of giving a high confidence “signal absent” response and decreasing the likelihood of giving a high‐confidence “signal present” response. (B). The figure illustrates the effect on metacognitive bias shift following continuous Theta Burst Stimulation (cTBS) over the right parietal cortex. After stimulation, participants showed less modulation of their alpha rhythm in response to the probabilistic cues. This lack of alpha modulation attenuates the shift of internal response distributions, thereby reducing the participants' metacognitive bias shift.

At the computational level, our findings can be interpreted within the metacognitive extension of SDT [9, 10]. In this framework, confidence judgments depend on the magnitude of the internal response reflecting the strength of sensory evidence [63]. Confidence is assigned by comparing this response to two criteria: one for confidence in signal presence, the other for confidence in signal absence [10]. Within this model, a high‐confidence judgment is given when the internal response exceeds these criteria; otherwise, a low‐confidence judgment is provided (Figure 6). For example, the presentation of a target inducing a strong internal response exceeds the *meta‐criterion* and yields a high confidence judgment, while an ambiguous response would not exceed it, producing a low confidence report. Crucially, shifting the entire response distribution toward one threshold increases high‐confidence judgments on that side without altering metacognitive sensitivity. Here, we propose that these shifts in response distributions may originate from the cue‐induced modulations of prestimulus alpha‐band activity [50, 62]. According to gating‐by‐inhibition models, alpha rhythms regulate the gain of sensory input by modulating cortical excitability: lower vs. higher alpha power increases vs. decreases excitability, thereby amplifying vs. attenuating the neural representation of incoming evidence [50, 64]. Consistent with this account, high‐probability cues elicited a transient suppression of posterior alpha amplitude prior to stimulus onset, consistent with increased excitability that results in a rightward shift in the internal response distributions, biasing judgments toward high‐confidence “signal present” responses. In contrast, low‐probability cues enhance alpha amplitude, reducing excitability and shifting the distribution toward the “signal absent” confidence boundary. On this interpretation, cue‐induced alpha fluctuations may operate as a multiplicative gain mechanism, systematically steering the internal response distribution toward one *meta‐criterion* or the other [62].

Data‐driven analyses showed that the relationship between expectation‐driven alpha modulation and *metacognitive bias shift* was strongest over parietal channels. This spatial pattern aligns with a growing body of evidence implicating the parietal cortex in metacognitive processing and in the integration of prior information in perceptual decision‐making [22, 39, 40, 46, 47]. For example, Park and colleagues [65] have recently demonstrated that confirmation biases arise not from altered sensory encoding but from a selective utilization of choice‐consistent evidence encoded in posterior parietal cortex. While their findings concern how prior choices bias the weighting of subsequent sensory evidence, our results reveal a complementary mechanism operating at the metacognitive level: externally cued expectations modulate parietal alpha amplitude, which in turn shifts the internal response distributions, biasing confidence judgments in line with prior probability. This interpretation is bolstered by previous causal evidence showing that the parietal cortex exerts control over posterior alpha rhythms [41, 43]. Extending this evidence, our results offer neurophysiological support for the idea that parietal regions serve as convergence zones where predictive and sensory signals are integrated via alpha dynamics to inform confidence.

Crucially, to test the causal role of parietal alpha‐mediated gain control in shaping confidence judgments, we conducted a second experiment in a cohort of 88 participants in which continuous Theta‐Burst Stimulation (cTBS) was applied over right parietal cortex (rPC) [66, 67] to transiently disrupt alpha dynamics and assess their impact on *metacognitive bias shift*. We demonstrated that inhibiting parietal activity through cTBS led to a selective attenuation of the cue‐induced modulation of pre‐stimulus alpha oscillations. This neurophysiological disruption was mirrored in behavior: participants' *metacognitive bias shift* induced by expectation cues was reduced. Specifically, the characteristic congruency effect observed in Study 1, where confidence increased when perceptual responses aligned with expectations and decreased when they did not, was attenuated.

Our findings suggest that parietal activity is not merely correlated with, but necessary for embedding prior beliefs into confidence judgments. Consistent with this interpretation, mediation analysis revealed a significant indirect effect, indicating that cue‐induced alpha modulation statistically mediates the stimulation‐related reduction in *metacognitive bias shift*. Importantly, this indirect effect emerges specifically in relation to the active stimulation (cTBS vs SHAM), supporting the specificity of the effect. Within the metacognitive SDT framework introduced above, these results further suggest that disrupting parietal function attenuates the alpha‐mediated gain mechanism that produces cue‐induced shifts in the internal response distributions. As a result, confidence judgments become less sensitive to prior information, reducing the *metacognitive bias shift*. These results could also be framed within the predictive coding framework [1], with this effect reflecting a breakdown in precision‐weighted inference: following parietal activity suppression, alpha dynamics become less sensitive to probabilistic cues, weakening the influence of expectations and shifting confidence toward bottom‐up evidence.

The selective reduction in *metacognitive bias* following cTBS, without concomitant changes in metacognitive sensitivity, complemented the pattern observed in Study 1 and aligned with our hypothesis. This selectivity was further corroborated by control analyses showing that cTBS affected neither overall confidence levels nor cue‐independent alpha amplitude, ruling out a non‐specific baseline shift account. This finding is also in line with recent causal evidence showing that inhibitory neurostimulation of the parietal cortex selectively disrupts spatial choice bias during endogenous attention tasks [68]. Although their focus was on attentional reorienting, the pattern of bias modulation paralleled our metacognitive results. Taken together, these studies suggest that PC may contribute to bias regulation across different cognitive domains, spanning both spatial orienting and confidence judgments. It is worth noting, however, that under conditions in which prior knowledge affects both metacognitive bias and metacognitive sensitivity [28], the parietal cortex might contribute to integrating expectations into both components. In this framework, the parietal cortex might operate as a dynamic weighting system, calibrating the relative impact of expectations on confidence judgments according to the task at hand.

A related literature shows that spontaneous pre‐stimulus alpha power predicts subjective visibility/awareness independently of sensitivity [69, 70, 71]. This dissociation is conceptually aligned with our finding that expectations modulate metacognitive bias without altering metacognitive sensitivity. However, our results reveal a distinct signature: confidence depends on prior‐response alignment, increasing when responses match expectations and decreasing when they contradict them, including elevated confidence for “absent” responses under low‐probability cues, when alpha amplitude is relatively high (Figure 6). Since visibility measures such as PAS (Perceptual Awareness Scale) are substantially lower on catch trials than on target‐present trials [69, 70], and high alpha power is associated with reduced subjective visibility, a visibility‐only account is difficult to reconcile with high confidence for stimulus‐absent reports in low‐excitability states.

While these findings support a causal contribution of the parietal cortex in expectation‐driven *metacognitive bias*, the experimental design does not allow us to determine whether analogous effects generalize to the left hemisphere. Future studies could therefore systematically vary both the stimulated hemisphere and the lateralization of stimulus presentation (e.g., left parietal stimulation with right visual field stimuli) to provide a more comprehensive evaluation of hemispheric effects. Including an active control site in the contralateral parietal cortex, or a functionally distinct control site, would further enhance this approach, allowing future work to directly compare stimulation over the target region with alternative sites and thereby clarify the neural mechanisms underlying the observed effects. Moreover, our findings speak specifically to the mechanisms underlying metacognitive bias in perceptual decision‐making, and their implications may not extend to other metacognitive domains.

Together, these findings reveal that confidence judgments are not passively read out from sensory evidence but are actively constructed through the integration of internal predictions. We show that prior expectations shape confidence judgments via modulations of posterior alpha rhythms, and that disrupting parietal activity with cTBS reduces this bias. More broadly, parietal alpha modulation may operate as a gain control mechanism supporting metacognitive self‐consistency by tuning sensory excitability, such that confidence judgments remain anchored to contextual expectations. This regulatory role, by revealing how the brain calibrates confidence according to prior information, offers a mechanistic entry point to understand, and ultimately modulate, confidence computations in both healthy cognition and neuropsychiatric conditions.

## 4 Methods

### 4.1 Participants

#### 4.1.1 Study 1

Seventy‐five healthy participants (39 female) took part in the study. This sample was drawn from a previously published dataset [23].

#### 4.1.2 Study 2

Eighty‐eight healthy participants (56 female) took part in the study. Participants were randomly assigned to the experimental group and the control group and received the cTBS (44 participants) or SHAM (44 participants) protocol, respectively. Both groups were matched for sample size and gender.

In both studies, participants were aged between 18 and 35 years. All participants provided informed consent before taking part in the study, which was conducted in accordance with the Helsinki Declaration and received approval from the Bioethics Committee of the University of Bologna (protocol code 201723, approved on 26 August 2021). None of the participants had a clinical history of neurological or psychiatric disorders, nor any conditions that would contraindicate the use of TMS (according to Rossi and colleagues [72]).

### 4.2 Stimuli and Experimental Task

The stimuli were presented on an 18″ CRT monitor (Cathode Ray Tube, CRT) with a display resolution of 1280 × 1024 pixels and a refresh rate of 85 Hz. They consisted of checkerboards presented in the lower‐left visual field (Figure 1A). Each cell of the checkerboards could contain small gray circles (target) or not (catch trials). The stimuli were created and presented using MATLAB (version 2016, The MathWorks Inc., Natick, MA) in combination with the Psychophysics Toolbox [73]. Participants were instructed to indicate, as quickly and accurately as possible, the presence or absence of gray circles within the checkerboard using the “K” and “M” keys on the keyboard. Additionally, they were asked to respond with their right hand. Throughout the task, participants were instructed to maintain their gaze on a fixation cross displayed at the center of the screen.

Each participant completed an adaptive titration procedure to determine the contrast of the gray circles (perceptual threshold) required to achieve a target detection accuracy of ∼70%. The overall accuracy in the main task was approximately 70% (namely, 71.9%), confirming the effectiveness of the titration phase. This procedure was conducted by presenting to the participant an equal number of trials with the target present and trials with the target absent (catch trials). The latter were included to prevent potential confounding effects caused by Type I bias; in fact, presenting only trials with the target present would not allow us to determine whether differences in threshold values were due to actual perceptual ability or to a different decision‐making style [74, 75, 76].

During the test phase (Figure 1A), each block consisted of 90 trials. At the beginning of each trial, a cue indicating the probability of target presence was displayed at the center of the screen for 1 s, followed by a fixation cross. The cue consisted of a rectangle with its lower portion colored red and the upper portion colored blue. The percentage of red indicated the probability of the target being present. Cue high and cue low (informative cues) indicated the probability of the presence of the target of 67% and 33%, respectively. Instead, the neutral cue (un‐informative cue) equally predicted (50%) the presence and absence of the target. The probability of target presentation was in accordance with the one indicated by the cue. Participants were also explicitly told that the probabilistic cue was congruent with the actual probability of stimulus presentation. After a variable interval of 1.2–1.5 s, a checkerboard appeared in the lower‐left quadrant of the screen (with or without the gray circles) for 60 ms. At this stage, the contrast of the gray circles was set to the threshold intensity identified through the adaptive titration procedure. The stimulus was presented in the left hemifield to prevent interference in the results due to spontaneous variations in attention between hemifields. After the participant provided their response, they were asked to indicate their confidence level regarding the response just given. Confidence was rated using the keyboard keys “1”, “2”, “3”, and “4” corresponding, respectively to four levels of confidence: not confident at all (1), slightly confident (2), moderately confident (3), and highly confident (4).

### 4.3 Experimental Procedure

Participants were seated in a dimly lit room in front of a monitor positioned at a distance of ∼ 57 cm. The EEG cap was then fitted according to the international 10–10 system. Initially, participants were familiarized with the task through a training phase, and each participant's individual perceptual threshold was determined using an adaptive titration procedure.

#### 4.3.1 Study 1

Participants were required to complete 6 blocks of the experimental task (Figure 1A).

#### 4.3.2 Study 2

Participants completed 3 experimental blocks, after which the active motor threshold [77] was determined and the cTBS or SHAM stimulation protocol was applied, with participants being randomly assigned to one of the protocols. Finally, 5 min after the stimulation [78], participants performed 3 experimental blocks again (Figure 3A).

### 4.4 Type I Signal‐Detection Theory (SDT) Modeling

The Type I SDT measures *d*′ and *criterion* [79] were computed. *d*′ quantifies a participant's stimulus sensitivity, with higher values indicating greater discriminative ability. In contrast, *criterion* quantifies the subject's Type I bias. A *c* value different from 0 indicates the presence of a Type I bias. Specifically, a *c* value greater than 0 reflects a more conservative criterion (i.e., tendency to give “absent response”), while a *c* value smaller than 0 reflects a more liberal criterion (i.e., tendency to give “present response”).

### 4.5 Type II Signal‐Detection Theory (SDT) Modeling

The Type II SDT measures *meta‐d*′ and *meta‐criterion* [9, 10] were computed. *meta‐d*′ is a measure of metacognitive sensitivity expressed within the framework of Type I SDT. It can be understood as the amount of signal available to the observer for performing the confidence evaluation task (Type II task). The higher the *meta‐d*′, the greater the participant's metacognitive ability. An individual with optimal metacognitive sensitivity will always be more confident when correct and less confident when incorrect.

*Meta‐criteria*, on the other hand, represent Type II bias calculated within the *meta‐d*′ framework and indicate the tendency to give high or low confidence ratings. A *meta‐criterion* value close to 0 suggests a more liberal criterion (i.e., tendency to give high confidence ratings), while a *meta‐criterion* value farther from 0 indicates a more conservative criterion (i.e., tendency to give low confidence ratings).

### 4.6 Continuous Theta Burst Stimulation

Participants received either continuous Theta Burst Stimulation (cTBS) or SHAM stimulation. The type of stimulation each participant received was randomly assigned. Stimulation was delivered using a Magstim Rapid TMS machine with an eight‐shaped coil (diameter = 7 cm) and consisted of triplets of pulses at a 50 Hz frequency, administered every 200 ms (5 Hz) for a total duration of 40 s, resulting in 600 pulses overall. This procedure induces inhibitory effects on cortical excitability lasting ∼50 min after stimulation [66]. For each participant, the entire POST session was conducted within this time window. Stimulation intensity was scaled to 80% of each participant's active motor threshold (aMT), which was determined over the left M1, following similar procedures in prior TBS studies (e.g. [80, 81],). The aMT was defined as the minimum stimulation intensity (expressed as a percentage of the maximum output power of the Magstim device) that elicited a visible muscle contraction in the right hand in at least 3 out of 5 trials when a single pulse was applied to the left motor area. During this procedure, the participants maintained a slight muscle contraction in the hand contralateral to the stimulation side. For technical and safety reasons, the maximum stimulation intensity was set at 45% of the device's maximum output power. The mean aMT across participants was 58% of the maximum stimulator output (MSO). Because 80% of aMT exceeded the safety cap (45% MSO) for some participants, the mean delivered intensity (43.6% MSO) was slightly lower than the mean intended intensity (46.4% MSO). This intensity range is consistent with previous studies targeting posterior parietal and parieto‐occipital regions (e.g., [40, 82, 83, 84, 85, 86, 87]). Stimulation was applied over electrode site P4 in line with the results from the brain‐to‐behavior analysis conducted in Study 1, which showed that P4 was the electrode showing the strongest correlation between Metacognitive bias modulation and alpha amplitude modulation (see results of Study 1 for details). For SHAM stimulation, the same protocol was followed, but the coil was positioned perpendicularly to the scalp to prevent any actual neurophysiological effects. Participants were asked to indicate the stimulation condition they thought they had received (active, SHAM, or uncertain); a chi‐square test revealed no significant differences between groups (X^2^ = 0.83, p = 0.66), indicating that participants could not reliably distinguish between active and SHAM conditions.

### 4.7 EEG Preprocessing and Time‐Frequency Decomposition

#### 4.7.1 Study 1 and Study 2

Participants sat comfortably in a room with dimmed lights. A set of 64 electrodes was mounted according to the international 10–10 system. EEG signals were acquired at a rate of 1000 Hz and all impedances were kept below 10 kΩ. EEG was processed offline with custom MATLAB scripts (version R2022b) and with the EEGLAB toolbox [88]. The EEG recording was filtered offline in the 0.5–100 Hz band and a notch‐filter at 50 Hz was applied. The signals were visually inspected, and noisy channels were spherically interpolated. Epochs spanning −4100 to 2000 ms relative to checkerboard onset were extracted and individual trials were visually inspected and those containing excessive noise, muscle or ocular artifacts were discarded. Next, the recording was re‐referenced to the average of all electrodes, and the Independent Component Analysis (ICA) was applied, an effective method largely employed for removal of EEG artifacts. Components containing artifacts that could be clearly distinguished from brain‐driven EEG signals were subtracted from the data. After these steps, the signals were downsampled to 256 Hz and a Laplacian transform was applied to the data using spherical splines. The Laplacian is a spatial filter that aids in topographic localization by attenuating artifacts attributable to volume conduction [89]. Subsequently, time‐frequency analysis was implemented by convolving the time series data with a set of complex Morlet wavelets (whose number of cycles increased from 5 to 13 as a function of frequency), defined as complex sine waves tapered by a Gaussian. Convolution was performed via frequency‐domain multiplication, in which the Fourier‐derived spectrum of the EEG data was multiplied by the spectrum of the wavelet, and the inverse Fourier transform was taken. Then, the amplitude was obtained by extracting the absolute value of the resulting complex time series. Amplitude was then condition‐specific baseline‐corrected using a decibel (dB) transform: dB amplitude = 10 × log10 (amplitude/baseline). Baseline amplitude was defined as the average amplitude in the period ranging from −3100 to −2700 ms before stimulus onset. Baseline correction (dB transform) was applied to normalize inter‐individual differences in absolute amplitude and to express changes on a scale comparable across frequencies; the baseline window (−3100 to −2700 ms, fixation‐only) was selected to precede cue onset and thus avoid contamination by expectation‐related activity.

### 4.8 SDT Type I Analysis

#### 4.8.1 Study 1

To examine the influence of the probabilistic cue on Type I sensitivity and Type I bias, d′ (*Z*(*Hit* *rate*) − *Z*(*False* *Alarm* *rate*)) and criterion (− 1/2 * [*Z*(*Hit* *rate*) + *Z*(*False* *Alarm* *rate*)]) were calculated separately for trials preceded by the high‐, low‐, or neutral‐probability cues (Figure 1B). Repeated‐ measures ANOVA were conducted with cue type as a within‐subject factor (three levels: high‐, low‐, and neutral‐probability) to statistically assess cue‐related effects on Type I sensitivity and Type I bias. Post‐hoc paired‐sample *t*‐tests were performed to interpret the ANOVA results, with p‐values corrected for multiple comparisons.

#### 4.8.2 Study 2

To investigate potential differences related to the stimulation received, *d*′ and *criterion* were computed separately for trials preceded by the high‐, low‐, or neutral‐probability cues, considering both stimulation protocol (cTBS, SHAM) and sessions (PRE, POST; see “Supporting Information”). To examine these effects, a three‐way repeated‐measures ANOVA was conducted with cue type (three levels: high‐, low‐, neutral‐probability) and sessions (two levels: PRE, POST) as within‐subject factors, and stimulation condition (two levels: cTBS, SHAM) as a between‐subject factor. Post‐hoc paired‐sample *t*‐tests were performed to interpret the ANOVA results, with p‐values corrected for multiple comparisons.

### 4.9 SDT Type II Analysis

#### 4.9.1 Study 1

To evaluate the effect of the probabilistic cue on metacognitive sensitivity (*meta‐d′)* and metacognitive bias (*meta‐criterion), meta‐d′ and meta‐criteria* were calculated separately for trials preceded by a cue indicating high‐, low‐, or neutral‐probability of target presence (Figure 1C,E). The *meta‐d*′ and *meta‐criteria* values were extracted using the Hierarchical *meta‐d*′ approach (*HMeta‐d*′) [44]. The analysis was performed using the hierarchical fitting option available in the *HMeta‐d*′ toolbox. This approach involves a Bayesian hierarchical estimation of the parameters, which returns the most probable value of the parameter in each condition. Specifically, this method integrates Bayesian priors to constrain estimates of both the group's average metacognitive sensitivity and individual efficiency using hierarchical modeling. The Bayesian hierarchical estimation implemented in *HMeta‐d*′ specifies prior density distributions at the group level for each participant‐level parameter and provides a group‐level estimate of metacognitive sensitivity (*meta‐d*′). Group‐level fits were performed using the function *fit_meta_d_mcmc_group. *Importantly, whenever we report or use individual estimates of *meta‐criterion* (e.g., for brain–behavior correlations), these values were obtained from the participant‐level posterior distributions returned by the hierarchical fit, rather than from independent single‐subject fits. This choice was motivated by simulation evidence showing that, when trial counts per participant/condition are limited, hierarchical estimation yields more reliable individual parameters and improves sensitivity for individual‐differences analyses without systematically biasing parameter‐trait correlations [90].

To analyze the effect of the cue on metacognitive sensitivity, *meta‐d*′ was examined by first computing the distribution of differences in the posterior parameter samples for each cue (high‐, low‐ or neutral‐probability cue) and then determining the 95% Highest Density Interval (HDI) for this distribution. The group‐level posterior densities were then used to test the statistical significance of differences in metacognitive sensitivity. Specifically, if the 95% HDI of the difference between conditions did not include 0, the difference was considered credible, whereas if the HDI included 0, the difference was deemed non‐significant.

Subsequently, to analyze the effect of the probabilistic cue on *meta‐criterion*, the same methodology was used as in the previous analyses. *meta‐criterion* was calculated separately for high‐, low‐, and neutral‐probability cues and separately for each Type I response (present/absent), resulting in six distinct *meta‐criterion* values. Finally, to examine the effect of the cue on *meta‐criterion*, a general index called metacognitive bias shift was computed (Figure 1D). This index was derived by summing two differences: the difference between the *meta‐criterion* value for absent responses in trials preceded by the low‐probability cue (LpA) and in trials preceded by the high‐probability cue (HpA), and the difference between the *meta‐criterion* value for present responses in trials preceded by the low‐probability cue (LpP) and in trials preceded by the high‐probability cue (HpP).

$$\begin{array}{ccc} & & \left(\mathrm{Meta}\mathrm{cogn}\mathrm{itive}\;\mathrm{bias}\;\mathrm{shift}\right.\\ & & =\left.\left[\mathrm{Meta}\_c_{\mathrm{LpA}}-\mathrm{Meta}\_c_{\mathrm{HpA}}\right]+\left[\mathrm{Meta}\_c_{\mathrm{LpP}}-\mathrm{Meta}\_c_{\mathrm{HpP}}\right]\right). \end{array}$$

A greater value suggests a larger shift in bias for congruent responses vs. incongruent ones.

Finally, because under the HMeta‐d′ parameterization *meta‐criteria* are expressed relative to the *criterion*, we performed an additional analysis using the m‐distance metric [45], which expresses the absolute distance between the *meta‐criteria* and the *criterion*, normalized by metacognitive sensitivity (*meta‐d')*. Thus, it allowed us to test whether the cue‐induced modulation of metacognitive bias was preserved after controlling for Type I criterion shifts. For each cue condition, m‐distance was computed separately for Type I “present” and “absent” responses (denoted rS2 and rS1, respectively) using the formula provided by Sherman et al.:

$$m\_\mathrm{dist}\mathrm{anc}e_{\mathrm{rS}1}=\left(\mathrm{meta}\_c\;-\;\mathrm{meta}\_c2_{R1}\right)\;/\;\mathrm{meta}\_d^{\prime }$$

$$m\_\mathrm{dist}\mathrm{anc}e_{\mathrm{rS}2}=\left(\mathrm{meta}\_c2_{R2}-\;\mathrm{meta}\_c\right)\;/\;\mathrm{meta}\_d^{\prime }$$

Larger values indicate a more conservative confidence policy (i.e., *meta‐criteria* placed farther from the *criterion*, requiring stronger evidence to report high confidence), whereas smaller values indicate a more liberal confidence policy. To follow a similar logic of our main metacognitive‐bias metric, we then computed a global m‐distance bias shift as:

$$\begin{array}{ccc} m\_\mathrm{dist}\mathrm{ance}\;\mathrm{bias}\;\mathrm{shift} & = & \left(m\_\mathrm{dist}\mathrm{anc}e_{\mathrm{HpA}\;}-\;m\_\mathrm{dist}\mathrm{anc}e_{\mathrm{LpA}}\right)\\ & & +\left(m\_\mathrm{dist}\mathrm{anc}e_{\mathrm{LpP}}\;-\;m\_\mathrm{dist}\mathrm{anc}e_{\mathrm{HpP}}\right) \end{array}$$

where *LpA/HpA* refer to “absent” responses following low/high‐probability cues and *LpP/HpP* to “present” responses following low/high‐probability cues. Posterior contrasts between conditions were evaluated by computing the posterior distribution of pairwise differences and reporting the 95% highest density interval (HDI); effects were considered credible when the HDI excluded zero.

#### 4.9.2 Study 2

To investigate whether the effect of the probabilistic cue on metacognitive sensitivity and metacognitive bias differed depending on the stimulation condition, *meta‐d*′ and *metacognitive bias shift* were calculated separately before and after the two stimulation conditions (cTBS, SHAM; Figure 3B,C). As in Study 1, participant‐level *meta‐d*′ and *meta‐criterion* values (when needed for subject‐level plots or correlations) were extracted as summaries of the participant‐level posteriors from the hierarchical fits. Because Study 1 showed that probabilistic cues did not modulate metacognitive sensitivity (*meta‐d'*), the cue factor was collapsed for the analysis of this measure. The resulting *meta‐d'* distributions from the hierarchical model were then compared between cTBS and SHAM groups across PRE and POST sessions. Regarding the effect on metacognitive bias, Study 1 demonstrated that the modulation of the *meta‐criterion* emerged as a function of the congruence between cue‐induced expectations and perceptual responses rather than being specific to a particular response category (i.e., present vs. absent). Accordingly, we operationalized this congruency‐dependent modulation as a *metacognitive bias shift* (computed as described in Study 1) by collapsing across both responses. The index was computed separately for PRE and POST sessions in both stimulation groups (cTBS, SHAM). To assess stimulation‐specific changes in metacognitive bias, we computed the difference between PRE and POST distributions separately for each group (PRE − POST), where positive values indicate a reduction in *metacognitive bias* following stimulation. To directly compare the two groups, we then computed the between‐group difference (cTBS − SHAM) separately for PRE and POST sessions, and finally the interaction contrast ([cTBS_PRE − SHAM_PRE] − [cTBS_POST − SHAM_POST]). For all contrasts, effects were considered credible when the 95% HDI of the posterior difference excluded zero.

### 4.10 EEG Analysis—Oscillatory Amplitude

#### 4.10.1 Study 1

To assess whether there is a relationship between pre‐stimulus oscillatory activity and the behavioral modulations induced by the expectancy cue, an amplitude analysis on a cluster of central and right posterior electrodes was conducted, given that the visual stimuli were presented in the left visual field (Figure 2A). Specifically, the mean amplitude was computed across the following electrodes: O2, PO4, PO8, P2, P4, P6, P8. This cluster was selected to capture contralateral posterior activity while minimizing dependence on single‐electrode selection. To examine pre‐stimulus oscillatory activity associated with prior integration, a non‐parametric permutation test based on a time × frequency cluster analysis (n = 1000) was performed on the amplitude difference between high‐ and low‐probability trials, within a 500‐ms pre‐stimulus window, spanning a broad frequency range (4–40 Hz). For each permutation, trial types were shuffled within each participant, generating a null distribution of amplitude differences. This data‐driven method allowed us to test, point by point, for significant differences between the two prior conditions across the entire time window and for all included frequencies while controlling for multiple comparisons [91]. Subsequently, a topographical analysis was performed to examine the spatial distribution of the observed pre‐stimulus effects. This involved contrasting high‐ versus low‐probability cue trials for each electrode in the alpha frequency range (∼7–14 Hz) within a 500‐ms pre‐stimulus interval. A topographical cluster‐based permutation was performed (Figure 2A), applying the non‐parametric Wilcoxon signed rank test [92] to analyze spatially specific cue‐induced modulation of alpha activity over right parieto‐occipital electrodes. For each permutation, trial types were shuffled within each participant with the same shuffle for each electrode.

#### 4.10.2 Study 2

To assess whether the relationship between pre‐stimulus oscillatory activity and the behavioral modulations induced by the expectancy cue differed based on the stimulation condition, the same EEG analysis of Study 1 was conducted separately for the two stimulation conditions (cTBS, SHAM) in both sessions (PRE, POST; Figure 4A,B). Moreover, to test the prediction that cTBS selectively reduces cue‐induced pre‐stimulus alpha modulation relative to SHAM, we computed for each participant a PRE–POST change score of the cue effect

$$\Delta \alpha =\alpha \mathrm{Ampl}\mathrm{itud}e_{\mathrm{CueL}\mathrm{owPr}\mathrm{obab}\mathrm{ility}\;}-\;\alpha \mathrm{Ampl}\mathrm{itud}e_{\mathrm{CueH}\mathrm{ighP}\mathrm{roba}\mathrm{bili}\mathrm{ty}}$$

within the a priori pre‐stimulus alpha ROI (–500–0 ms; ∼7–14 Hz), separately for cTBS and SHAM groups (shiftTBS, shiftSham). The interaction was then quantified as the difference between group means:

$$\Delta \Delta \alpha ={\left(\Delta \alpha_{\mathrm{PRE}}-\;\Delta \alpha_{\mathrm{POST}}\right)}_{\mathrm{cTBS}}-\;{\left(\Delta \alpha_{\mathrm{PRE}}-\;\Delta \alpha_{\mathrm{POST}}\right)}_{\mathrm{SHAM}}$$

and evaluated using a one‐tailed permutation test based on shuffling group labels (1000 permutations) and recomputing at each shuffle; the *p*‐value was computed as the proportion of permutations in which the permuted interaction statistic was greater than the observed interaction statistic, consistent with the directional hypothesis that cTBS produces a larger PRE–POST reduction than SHAM.

### 4.11 EEG Analysis—Brain‐To‐Behavior

#### 4.11.1 Study 1

To investigate the relationship between the behavioral modulations induced by the expectancy cue and the oscillatory modulations, a correlation analysis was conducted between *metacognitive bias shift* and the trial‐type amplitude difference (high‐ vs. low‐probability) at each time–frequency point within a 500‐ms pre‐stimulus window, across the 4–40 Hz band using MATLAB's *robustfit* function (Figure 2B). This approach downweights outliers rather than removing them, yielding more reliable estimates in the presence of influential observations. Each point in the time‐frequency map obtained from the previously described analysis was used as a predictor and correlated with the *metacognitive bias shift*. Time–frequency points with correlation values not surviving the cluster‐forming threshold were removed, and clusters were subsequently defined as groups of adjacent time–frequency points exhibiting significant correlations. Then a correction for multiple comparisons was run using a cluster‐based permutation approach. For each permutation, *metacognitive bias shift* values were shuffled across participants generating a null distribution of behavioral patterns. Finally, a topographical analysis was performed by extracting, for each electrode (time: –500–0 ms), the Δ alpha amplitude (∼7–14 Hz; i.e., the amplitude difference between high‐ and low‐probability trials in the alpha band) and correlating it with the *metacognitive bias shift* (Figure 2B). The alpha shift was selected because the significant cluster from the preceding data‐driven analysis was centered in the alpha range. This approach allowed us to identify the spatial distribution of the correlation across the scalp and to determine the electrode where the relationship was strongest. To investigate this, we performed robust regressions between the prestimulus oscillatory amplitude difference (high vs. low probability) and individual *metacognitive bias shift* at each electrode. For each regression, we extracted the t‐statistic of the slope (i.e., the estimate divided by its robust standard error). The electrode showing the largest absolute t‐statistic was considered to exhibit the strongest correlation. Then, a correction for multiple comparisons was run using a cluster‐based permutation approach. Finally, for each participant we extracted the pre‐stimulus alpha shift from the parieto‐occipital ROI and related it to the *metacognitive bias shift* using robust linear regression.

#### 4.11.2 Study 2

To assess the replicability of the brain–behavior association identified in Study 1, we performed the same analysis on PRE‐session data from Study 2, pooling cTBS and SHAM participants at baseline (N = 88). Because Study 2 included a between‐subjects factor, we fitted a robust regression with effect coding (−0.5 = SHAM, +0.5 = cTBS) including the neural predictor, group, and their interaction. The main effect was tested using cluster‐based permutation (1000 iterations, stratified by group). The interaction was evaluated on the cluster‐averaged neural predictor extracted from the significant main‐effect cluster.

To further test the role of alpha oscillatory activity in driving the effect of stimulation type on changes in *metacognitive bias shift*, we conducted a simple mediation analysis using the PROCESS module for JASP [93], specifying Model 4 (Figure 5). Since cue‐induced alpha modulation was positively correlated with *metacognitive bias shift* in Study 1, it was expected that its suppression would be accompanied by a corresponding decrease in bias. The experimental group was coded as a binary predictor (0 = SHAM, 1 = cTBS). The mediator was the PRE‐to‐POST change in cue‐induced pre‐stimulus alpha amplitude modulation. First, cue‐induced alpha modulation was computed separately for each session as alpha shift = alpha amplitude_low‐probability cue − alpha amplitude_high‐probability cue within the pre‐stimulus alpha ROI. Then, the change in alpha modulation was computed as Δ alpha amplitude = alpha shift_PRE − alpha shift_POST. Thus, positive Δ alpha amplitude values indicate a reduction in cue‐induced alpha modulation from PRE to POST.(). The outcome was the PRE‐to‐POST change in *metacognitive bias shift* (Δ *metacognitive bias shift*; PRE − POST). Thus, positive values indicate a reduction from PRE to POST‐stimulation. Path coefficients a (X→M), b (M→Y controlling for X), the direct effect c′ (X→Y controlling for M), and the total effect c (X→Y) were estimated using regression, and the indirect effect was computed as the product a×b. Inference on mediation was based on bias‐corrected bootstrap confidence intervals for the indirect effect (1000 resamples). Because we had an a priori directional hypothesis (cTBS would produce a larger PRE‐to‐POST reduction in cue‐induced alpha modulation, and this reduction would be accompanied by a decrease in *metacognitive bias shift*), we evaluated the indirect effect using a one‐sided criterion: mediation was considered supported when the bootstrap confidence interval for a×b excluded zero in the hypothesised direction.

### 4.12 Statistical Analysis

All analyses were performed using MATLAB R2022b (MathWorks), JASP [93], the HMeta‐d' toolbox [44] and the EEGLAB toolbox [88]. Sample sizes were N = 75 (Study 1) and N = 88 (Study 2; n = 44 cTBS, n = 44 SHAM). The present work adopts a two‐stage analytical framework. Study 1 was used to identify robust effects and specify directional hypotheses for Study 2. In Study 1, frequentist tests were evaluated two‐tailed at α = 0.01. Study 2 then tested these hypotheses in an independent sample. Accordingly, only analyses with explicit a priori directional predictions derived from Study 1 were evaluated one‐tailed (α = 0.05), whereas baseline‐equivalence tests and analyses without directional predictions were evaluated two‐tailed (α = 0.05). Bayesian estimates are reported as posterior means with 95% HDI; in both studies, effects were considered credible when the HDI excluded zero. Frequentist results are reported as test statistics with exact *p*‐values.

## Author Contributions

Conceptualization: L.T. and V.R.; Methodology: L.T., D.R., and A.P.; Investigation: L.T., M.C., D.R., and A.P.; Visualization: L.T., A.P., and D.R.; Supervision: VR. and L.T.; Writing – original draft: L.T., A.P., D.R., and V.R.; Writing – review and editing: V.R., M.C., L.T, A.P., and D.R.

## Funding

VR is supported by the Next Generation EU (NGEU) and funded by the Ministry of the University and Research (MUR), National Recovery and Research Plan (NRRP) PRIN 2022 (grant no. 2022H4ZRSN—CUP J53D23008040006): Predictive waves in human perception and individual differences along the autism‐schizophrenia continuum (D DN. 104 02.02.2022); (grant no. P2022XAKXL—CUP J53D23017340001): Investigating the plasticity of human predictive coding through neuromodulation (D DN. 1409 14.09.2022); Ministerio de Ciencia, Innovación y Universidades, Spain (PID2019‐111335 GA‐100); LT is supported by Bial Foundation (241/24).

## Ethical Statement

The Study was Approved by the Bioethics Committee of the University of Bologna (Protocol Code 201723). The written informed consents were obtained from all the participants.

## Conflicts of Interest

The authors declare no conflicts of interest.

## Supporting information




**Supporting File**: advs75775‐sup‐0001‐SuppMat.docx.

## Data Availability

The data that support the findings of this study are available from the corresponding author upon reasonable request.
