# Supplementary material for: Prior Expectations Bias Confidence Judgments Through Parietal Alpha‐Band Modulation
Source: Adv Sci (Weinh). 2026 May 23;13(46):e19417. doi: 10.1002/advs.202519417 (PMC13336109; doi:10.1002/advs.202519417)
Supplement: Supplementary file 1 [file ADVS-13-e19417-s001.docx]

**SUPPLEMENTARY INFORMATION**

***Figure S1. Type I Signal detection theory***

*Type I sensitivity (d’) and Type I bias (criterion) indices are represented separately for trials preceded by high- (in red) low- (in blue), or neutral-probability (in green) cues in PRE and POST sessions for both SHAM and cTBS conditions. The Y-axis represents the values of the two parameters, and each circle corresponds to a subject. Crucially, no main effects of stimulation or session were found for either d’ or criterion, nor were there any significant interactions between stimulation, sessions, and cue (all p > 0.14).*

**M-distance confirms cue-induced modulation of metacognitive bias independently of criterion shifts**

To address the concern that shifts in *criterion* (*c,* i.e. Type I bias) could propagate to *meta-criteria* estimates under the HMeta-d′ parameterization, we ran a control analysis using as dependent variable the m-distance metric (Sherman et al., 2018), which quantifies the normalized distance between *meta-criteria* and the *criterion* (Type I bias; see Methods). For Type I “present” responses (rS2), m-distance was credibly smaller for high probability cue (mean = 0.61) than low probability cue (mean = 0.80; HDI_low-high_ [0.14, 0.25]), indicating a more liberal metacognitive bias when the perceptual response was congruent with the high-probability cue. For Type I “absent” responses (rS1), m-distance was credibly larger for high probability cue (mean = 0.62) than low probability cues (mean = 0.40; HDI_low-high_ [−0.26, −0.17]), indicating a more conservative metacognitive bias when the perceptual response was incongruent with the high-probability cue. Thus, the same cue induced opposite shifts in metacognitive bias depending on whether the Type I response aligned with the prior expectation, reproducing the congruence-dependent pattern reported in the main analysis. Finally, we computed an m-distance bias shift analogous to the main *metacognitive bias shift*, capturing the overall cue-dependent modulation of *meta-criteria* while controlling for *criterion* (Type I bias) shifts. This analysis revealed a robust effect: the m-distance bias shift was greater than zero (mean = 0.41; HDI [0.34, 0.49]), indicating that probabilistic cues modulate metacognitive bias even after removing any contribution of *criterion* (Type I bias) shifts. Together, these findings indicate that expectation-related modulation of metacognitive evaluation is preserved even when quantified using a measure explicitly independent of *criterion* (Type I bias) shifts.

**Figure S2. Cue-induced changes in m-distance replicate the main metacognitive bias pattern while controlling for criterion (Type I bias) shifts.**
m-distance^[44]^ quantifies the normalized distance between *meta-criteria* and the *criterion* (Type I bias), controlling for shifts in *criterion* (Type I bias). (A) For “present” responses (rS2), m-distance was credibly smaller for high probability cue (mean = 0.61) than low probability cue (mean = 0.80; HDI_low-high_ [0.14, 0.25]) indicating a more liberal metacognitive bias when responses are congruent with the cue. (B) For “absent” responses (rS1), m-distance was credibly larger for high probability cue (mean = 0.62) than low probability cues (mean = 0.40; HDI_low-high_ [−0.26, −0.17]), indicating a more conservative metacognitive bias when responses are incongruent with the cue.(C) Posterior distribution of the m-distance bias shift (defined analogously to the main metacognitive bias shift) showing a credible shift above zero (mean = 0.41; HDI [0.34, 0.49]).

**3D cluster-based permutation test across channels × time × frequency**

To corroborate the findings reported in the main text and address potential concerns regarding circularity, we conducted a three-dimensional cluster-based permutation test operating simultaneously in channel × frequency × time space. For each participant, time–frequency representations were computed at each of the 63 scalp electrodes, yielding a three-dimensional data structure [channels × frequencies × time] for each experimental condition. A subject-level contrast was computed by subtracting the two conditions of interest (high - low probability cue), and the group-level mean difference was converted into an empirical *z*-score map relative to a null distribution obtained via sign-flip permutation testing (1000 permutations). The analysis covered the 500 ms pre-stimulus window (−500 to 0 ms) across 40 logarithmically spaced frequencies from 4 to 40 Hz. To identify clusters, suprathreshold voxels were grouped based on three-dimensional adjacency: temporal adjacency, spectral adjacency, and spatial adjacency between electrodes. Critically, we implemented an overlap-gated spatial adjacency rule to prevent artificially inflated clusters. In standard 3D clustering, two spatially neighbouring electrodes can merge into the same cluster by sharing as few as one suprathreshold time–frequency pixel; a "thin bridge" that may arise by chance without reflecting genuine shared activity. To address this, we required that two adjacent electrodes share at least 10% of the total time–frequency pixels as simultaneously suprathreshold voxels before they could be connected. This threshold ensures that spatial merging reflects genuine spectrotemporal co-activation. The same overlap *criterion* was applied in every permutation iteration, guaranteeing that the null distribution was estimated under identical adjacency rules. Cluster significance was assessed by comparing observed cluster sizes against the null distribution of maximum cluster sizes. As a robustness check, we verified that significant clusters emerged in the same experimental conditions when the overlap constraint was removed entirely.

The analysis revealed significant clusters in all conditions except the POST session after cTBS. In Study 1, a significant negative cluster emerged centred in the alpha band (~7–14 Hz) over right parieto-occipital electrodes. In Study 2, significant clusters were observed in both PRE sessions (cTBS and Sham) and in the POST Sham session, all centred in the alpha band over parieto-occipital sites. No significant cluster emerged in the POST cTBS session, indicating a disruption of cue-dependent pre-stimulus alpha modulation following parietal cTBS. These results converge with the findings reported in the main text, demonstrating that the alpha-band effect and its disruption by cTBS are robust across analytical approaches.

**STUDY 1**

**STUDY 2**

**Figure S3. Spatial and spectral characterization of the significant 3D cluster (channels × frequency × time).** For each analysis, the left panel displays the spectral profile of the significant cluster, computed as the proportion of significant voxels (channels × time) within each frequency bin and normalized to a 0–1 scale. Shaded areas denote canonical frequency bands (θ, α, β₁, β₂, γ). In all cases where a cluster was detected, the spectral profile peaked within the alpha range, indicating that the effect was predominantly driven by alpha-band activity. The right panel shows the scalp distribution of the empirical z-values averaged across the pre-stimulus analysis window (–500 to 0 ms) and across all frequencies. Black dots mark electrodes belonging to the significant cluster after overlap-gated 3D cluster-based permutation correction.

From top to bottom:

**Study 1**. A significant negative cluster was observed, reflecting stronger pre-stimulus alpha suppression in high-probability relative to low-probability cue trials. The cluster spanned the entire pre-stimulus window, with a spectral peak in the alpha range (~7–14 Hz) and a right-lateralized parieto-occipital scalp distribution, consistent with the results obtained with the original two-step analysis.

**Study 2 – Sham (PRE and POST sessions).** Significant clusters were present prior to stimulation, reproducing the Study 1 pattern with alpha-centered clusters over right parieto-occipital electrodes. The right posterior cluster remained significant after stimulation, indicating that the cue-dependent modulation of cue-induced, pre-stimulus alpha modulation persisted in the absence of stimulation.

**Study 2 – cTBS (PRE and POST sessions).** Significant clusters were present prior to stimulation, reproducing the Study 1 pattern with alpha-centered clusters over right parieto-occipital electrodes. No significant cluster was detected after stimulation, indicating that parietal cTBS abolished the cue-dependent modulation of pre-stimulus alpha oscillations.

**Raw confidence analysis.**
To determine whether cTBS induced a general shift in confidence level rather than a selective effect on cue-induced metacognitive bias, we analysed mean raw confidence ratings collapsed across all experimental conditions. For each participant, we computed the mean confidence rating across all trials (collapsing across cue type) separately for the PRE and POST sessions. We then derived a within-participant change score: Δ = POST − PRE. A positive Δ indicates an overall increase in confidence from PRE to POST, while a negative Δ indicates a decrease. The critical question in this pre–post design with active and sham stimulation groups is whether the two groups show differential changes over time, operationalised as the Stimulation × Session interaction. This interaction can be quantified as a difference-in-differences: ΔΔ = Δ cTBS − Δ SHAM. To evaluate this interaction while making minimal distributional assumptions, we used a two-tailed permutation test with 1000 permutations. The null hypothesis is that group assignment (cTBS vs. SHAM) is exchangeable with respect to the change score Δ; that is, that cTBS and SHAM stimulation have equivalent effects on raw confidence. To this end, 1) we pooled all participants' change scores Δ, 2) randomly permuted the group labels (cTBS vs. SHAM), 3) recomputed ΔΔ as the difference between the mean change score of the two pseudo-groups, 4) repeat steps 2-3 for 1000 iterations to generate a null distribution of ΔΔ, 5) compute the *p*-value as the proportion of permutations where |ΔΔ permuted| ≥ |ΔΔ observed|. Critically, the Stimulation × Session interaction was not significant (observed ΔΔ = −0.033, *p* = 0.48). Together, these results indicate that cTBS did not produce an overall shift in confidence level, supporting the interpretation that stimulation selectively affects the cue-induced modulation of metacognitive bias.

**Figure S4. Mean confidence ratings are unaffected by parietal stimulation.** Mean confidence ratings collapsed across cue (and stimulus) conditions are shown for PRE and POST sessions in the SHAM and cTBS groups. Error bars indicate ±1 SEM across participants. The Stimulation × Session interaction on PRE–POST change scores was not significant (p = 0.48), indicating that cTBS did not induce a global shift in confidence level.

**Figure S5. Scatterplots illustrating the brain–behaviour association in Study 1 and Study 2.** Left panel: Study 1. Relationship between the pre-stimulus alpha shift extracted from the *a priori* parieto-occipital ROI (averaged across electrodes) and the *metacognitive bias shift*. Right panel: Study 2 (PRE, pooled Sham and cTBS participants). Relationship between the pre-stimulus alpha shift extracted from the same *a priori* parieto-occipital ROI and the metacognitive bias shift.

**Replication of the Brain-Behaviour Association in Study 2**

To assess the replication of the effect of the brain–behaviour association observed in Study 1, we pooled cTBS and Sham participants at baseline (combined *N* = 88). Because Study 2 included a between-subjects factor (stimulation group), we fitted a robust regression with effect coding (−0.5 = Sham, +0.5 = cTBS) at each analysis unit:

$$Metacognitive bias shift = b₁\cdot Neural + b₂\cdot Group + b₃\cdot(Neural \times Group)$$

where *b1* estimates the average brain–behaviour slope across groups and *b3* captures any difference in slope between groups. The main effect was tested using cluster-based permutation (1000 iterations; permutations stratified by group). The presence of an interaction was then evaluated on the cluster-averaged neural predictor extracted from the significant main-effect cluster to check whether the two groups showed a different brain-to-behaviour association.

We assessed replication along two complementary dimensions. A time–frequency cluster-based permutation analysis revealed a significant cluster centred in the alpha band, consistent with the spectrotemporal profile observed in Study 1. A complementary topographical analysis, in which alpha shift was averaged within the same time–frequency window of interest (~7-14 Hz, −500 to 0 ms) and tested independently at each electrode, revealed a significant spatial cluster over parieto-occipital sites, consistent with the spatial distribution observed in Study 1. In neither analysis was the neural × group interaction significant, indicating no evidence that the association differed between groups at baseline. Together, these findings show that the brain–behaviour association replicated in an independent sample in both its spectrotemporal and spatial characteristics.

**Figure S6. Replication of the brain–behaviour association in Study 2.**

Left panel: Time–frequency cluster-based permutation analysis of the brain–behaviour association in study 2. Colours represent the empirical regression statistics (t-values) across time and frequency, and black contours mark the significant cluster. We found a significant cluster that extended across the pre-stimulus window, matching the spectrotemporal profile observed in Study 1. Right panel: Topographical cluster-based permutation analysis of the association between cue-induced alpha shift and *metacognitive bias shift*. Alpha amplitude shift was averaged within the time–frequency window used in study 1, and the brain–behaviour association was tested independently at each electrode using robust regression. Colours represent the empirical regression statistics (t-values), and black dots indicate electrodes belonging to the significant spatial cluster, which was maximal over parieto-occipital sites. Across both analyses, the neural × group interaction was not significant, indicating no evidence that the brain–behaviour association differed between Sham and cTBS participants at baseline.

**Testing cue-independent PRE–POST changes and Stimulation × Session interaction**

The main EEG hypothesis tested in Study 2 targets a cue-dependent neural outcome (high–low cue contrast): cTBS is predicted to selectively attenuate the pre-stimulus alpha modulation by probabilistic cues relative to SHAM. To rule out the alternative explanation that stimulation induces a cue-independent (overall) shift in alpha amplitude from PRE to POST, we performed two control analyses. First, for each participant, we computed a cue-averaged time–frequency representation by averaging spectral amplitude across cue levels within each session (PRE, POST), using the same electrode set, time window, and frequency range defined a priori in the main analyses. This yields, for each subject and session, a cue-independent TF map reflecting overall pre-stimulus activation. Then, we tested cue-independent PRE–POST changes within each group separately using the same non-parametric cluster-based permutation framework adopted for the main TF analyses (paired comparison, identical time/frequency search space and clustering parameters). No significant PRE–POST clusters were observed in either group (SHAM: p = 0.16; cTBS: p = 0.54), indicating no robust cue-independent shift in pre-stimulus TF activations from PRE to POST within either stimulation condition. Second, we quantified the Stimulation × Session interaction on cue-averaged alpha amplitude as a difference-in-differences. For each participant we extracted mean alpha amplitude within the same a priori ROI (electrodes × time × frequency) from the cue-averaged maps in PRE and POST and computed a subject-level change score Δ = POST–PRE. The interaction statistic was then defined as:

$$\Delta\Delta= \Delta\text{cTBS} - \Delta\text{SHAM}$$

We evaluated ΔΔ against a permutation-based null distribution obtained by pooling participants’ Δ values across groups, randomly reassigning them to two pseudo-groups of the same sizes as the original cTBS and SHAM samples, and recomputing ΔΔ on each shuffle (1000 permutations). This control analysis demonstrated no interaction between Group and Time (observed ΔΔ = – 0.063; p = 0.76). Together, the absence of (i) cue-independent PRE–POST clusters within either group and (ii) a Stimulation × Session interaction on cue-averaged alpha amplitude indicates that parietal cTBS does not induce a global shift in pre-stimulus alpha activity. This supports the specificity of the main neural finding: the stimulation effect is expressed as a selective attenuation of cue-dependent alpha modulation, rather than as an overall change in alpha state.
